# Characterization and potential evolutionary impact of transposable elements in the genome of *Cochliobolus heterostrophus*

**DOI:** 10.1186/1471-2164-15-536

**Published:** 2014-06-28

**Authors:** Mateus F Santana, José CF Silva, Eduardo SG Mizubuti, Elza F Araújo, Bradford J Condon, B Gillian Turgeon, Marisa V Queiroz

**Affiliations:** Laboratório de Genética Molecular e de Micro-organismo, Universidade Federal de Viçosa, Viçosa, Brazil; Instituto Nacional de Ciência e Tecnologia em Interações Planta-Praga, Universidade Federal de Viçosa, Viçosa, Brazil; Departamento de Fitopatologia, Universidade Federal de Viçosa, Viçosa, Brazil; Department of Plant Pathology & Plant-Microbe Biology, Cornell University, Ithaca, USA

**Keywords:** Transposable elements, *Cochliobolus heterostrophus*, Repeat-induced point mutation, Genome

## Abstract

**Background:**

*Cochliobolus heterostrophus* is a dothideomycete that causes Southern Corn Leaf Blight disease. There are two races, race O and race T that differ by the absence (race O) and presence (race T) of ~ 1.2-Mb of DNA encoding genes responsible for the production of T-toxin, which makes race T much more virulent than race O. The presence of repetitive elements in fungal genomes is considered to be an important source of genetic variability between different species.

**Results:**

A detailed analysis of class I and II TEs identified in the near complete genome sequence of race O was performed. In total in race O, 12 new families of transposons were identified. *In silico* evidence of recent activity was found for many of the transposons and analyses of expressed sequence tags (ESTs) demonstrated that these elements were actively transcribed. Various potentially active TEs were found near coding regions and may modify the expression and structure of these genes by acting as ectopic recombination sites. Transposons were found on scaffolds carrying polyketide synthase encoding genes, responsible for production of T-toxin in race T. Strong evidence of ectopic recombination was found, demonstrating that TEs can play an important role in the modulation of genome architecture of this species. The Repeat Induced Point mutation (RIP) silencing mechanism was shown to have high specificity in *C. heterostrophus,* acting only on transposons near coding regions.

**Conclusions:**

New families of transposons were identified. In *C. heterostrophus,* the RIP silencing mechanism is efficient and selective. The co-localization of effector genes and TEs, therefore, exposes those genes to high rates of point mutations. This may accelerate the rate of evolution of these genes, providing a potential advantage for the host. Additionally, it was shown that ectopic recombination promoted by TEs appears to be the major event in the genome reorganization of this species and that a large number of elements are still potentially active. So, this study provides information about the potential impact of TEs on the evolution of *C. heterostrophus*.

**Electronic supplementary material:**

The online version of this article (doi:10.1186/1471-2164-15-536) contains supplementary material, which is available to authorized users.

## Background

*Cochliobolus heterostrophus* (anamorph, *Bipolaris maydis*) is the causative agent of Southern Corn Leaf Blight (SCLB) [1], a common disease of maize in tropical and subtropical regions [2–4]. *Cochliobolus* species belong to the class Dothideomycetes, order Pleosporales, which includes a large number of highly destructive plant pathogens [5]. *C. heterostrophus* is a necrotrophic fungus. There are two known races: race T, which produces a host selective toxin called T-toxin, and race O, which does not. In 1970, an SCLB epidemic caused by race T led to large economic losses in the eastern and southern United States. Currently, the disease is effectively controlled by planting resistant hybrids [4], but the pathogen remains an important subject of investigation as a model for mechanisms of pathogenicity and virulence as well as the evolutionary processes generating highly virulent strains. The ease with which the sexual cycle of *C. heterostrophus* can be induced in the laboratory and the development of efficient homologous integration techniques facilitate site-specific mutagenesis and make *C. heterostrophus* an excellent genetic model [6].

Race T is particularly virulent to plants that carry the Texas cytoplasmic male sterile trait (Tcms) as these plants are sensitive to T-toxin [7]. The ability to produce T-toxin is related to the presence of ~ 1.2-Mb of DNA encoding genes related to the production of T-toxin [8, 9]. Kodama et al. [10] detected two loci (*Tox1A* and *Tox1B*) related to the production of T-toxin. They found that these loci are associated with a translocation involving race O chromosomes 6 and 12. To date, nine genes responsible for the elevated virulence of race T have been identified. At the *Tox1A* locus, two genes (*PKS*) encode the polyketide synthases, *PKS1* [9] and *PKS2* [11], one gene each encodes *LAM1*, a putative 3-hydroxyacyl-CoA dehydrogenase [12], *OXI1*, a putative short-chain dehydrogenase [12], and *TOX9* [12] that has no predicted functional domains or homology to known genes. The *Tox1B* locus encodes the genes *DEC1*, a decarboxylase [13] and three reductases *RED1* [13], *RED 2,* and *RED3* [12]. None of these genes is found in race O. The production of T-toxin is complex, and the evolutionary origin of the associated genes is unclear. Additionally, the evolutionary process that led to the emergence of race T is not known [12, 14]. However, a large number of repeated sequences were found to flank *Tox1* [12] and a large proportion of repetitive tracts in the sequenced genomes share similarity with sequences for transposable elements (TEs). In fact, TEs have been associated with regions of virulence in various fungi including *Magnaporthe grisea* [15], *Fusarium oxysporum* [16] and *C. heterostrophus* race T itself [12].

Transposable elements can be classified in a hierarchical manner into: class, subclass, order, superfamily, family, and subfamily. There are two main classes of elements, distinguished by the presence or absence of an RNA intermediate. The elements of class I, retrotransposons, replicate through a “copy-and-paste” mechanism that generates RNA intermediates that are subsequently reverse transcribed into double-stranded DNA by enzymes encoded by TE DNA [17]. Each complete transposition cycle produces a new copy of the TE. Consequently, retrotransposons are frequently the major contributors to repetitive tracts in the genome. Retrotransposons can be divided into five orders based in the transposition mechanism, organization and phylogeny of the reverse transcriptase: LTR (Long Terminal Repeat) retrotransposons, DIRS-like (*Dictyostelium* Intermediate Repeat Sequence), Penelope-like, LINEs (Long Interspersed Nuclear Element), and SINEs (Short Interspersed Nuclear Element) [17].

LTR- and LINE-type retrotransposons are widely distributed in the genomes of fungi [18, 19]. The LTR superfamilies most commonly present in these genomes are *Gypsy* and *Copia*, which encode two regions known as *gag* and *pol*. The *gag* region encodes structural proteins that form a virus-like particle (capsid proteins). The *pol* region encodes a protease, a reverse transcriptase (RT), a RNAse and an integrase [20]. The *Gypsy* and *Copia* superfamilies differ in the order of genes encoding the RT and the integrase in the *pol* region [21]. LINEs lack long terminal repeat sequences, can vary in size, and have been separated into five superfamilies: *R2*, *L1*, *RTE*, *I,* and *Jockey*. Each superfamily is then divided into multiple families. Autonomous LINEs encode at least one RT and one nuclease in their *pol* ORF [open reading frame]. An ORF similar to the *gag* region is sometimes found in a position upstream of the *pol* region, but its role remains unclear. At the 3’ end of the LINEs, a poly(A) tail, tandem repeats or an A-rich region may be present [17]. The number of copies of non-LTRs varies enormously between the different sequenced fungal genomes [18].

Class II elements, the DNA transposons, are divided into two subclasses. Subclass I comprises elements that transpose by a mechanism of excision and integration, with both strands of DNA being cleaved during the excision process, while subclass 2 is composed of elements that duplicate themselves prior to insertion. Furthermore, subclass 1 comprises two orders, the most well-known being the TIR (Terminal Inverted Repeats). This order has nine superfamilies: *Tc1-Mariner*, *Mutator*, *hAT*, *Merlin*, *Transib*, *P*, *PIF/Harbinger*, *CACTA* and *Crypton.* Subclass 2 comprises also two orders: *Helitron* and *Maverick* [17]. There are also non-autonomous groups of TEs that lack one or more genes essential for transposition, including MITEs (Miniature Inverted-repeat Terminal Elements) in class II, SINEs in the non-LTR retrotransposons and TRIMs (Terminal-repeat Retrotransposon In Miniature) and LARDs (Large Retrotransposon Derivates) in the LTR retrotransposons [22].

Transposable elements are important in the generation of variability, structure and evolution of genomes [23]. Some of the effects of TEs may be due to ectopic recombination among TEs of the same family. Ectopic recombination occurs between homologous DNA sequences on different chromosomes (ie, identical TEs at two locations) and can have beneficial as well as potentially deleterious consequences for the eukaryotic genome. In general, however, the introgression of TEs into a genome is potentially harmful, as the activity of transposons and rearrangement sites can lead to a decrease in genome stability. As a result, many organisms have defense systems that repress the activity of TEs. One defense mechanism that has received particular attention is the Repeat-Induced Point Mutation (RIP) system. RIP is a gene silencing mechanisms that leads to the mutation of repetitive DNA sequences during the sexual cycle, between fertilization and nuclear fusion. In general, RIP induces GC-to-AT mutations in duplicated DNA sequences of more than 400 base pairs (bp) with sequence identity greater than 80% [18, 24]. Cytosine methylation is frequently associated with RIP-type mutations, and in *Neurospora crassa,* the methyltransferase (RID) is responsible for that methylation [25]. Evidence of RIP has been detected also in *C. heterostrophus* [5, 26].

Ohm et al. [5] analyzed 18 members of the class Dothideomycetes and found that genes encoding effector proteins frequently occur near TEs. The authors also showed that the action of the RIP silencing mechanism in sequences near TEs can expose those sequences to a high rate of point mutations. This phenomenon perhaps facilitates the response of the fungus to adverse environmental conditions and provides an advantage against its host. With the advent of genome sequencing, the analysis of TEs in genomes has become possible, particularly in model fungi such as *C. heterostrophus.* Therefore, due to the important role that transposons play in the evolutionary processes of this fungus, a broad search for and complete characterization of the major TEs distributed in the genome of *C. heterostrophus* was conducted. The results found in this study improve the understanding of the potential impact of TEs on the evolution of *C. heterostrophus*.

## Results

### Analysis of transposable elements in the genome of *C. heterostrophus*

**Figure 1:**
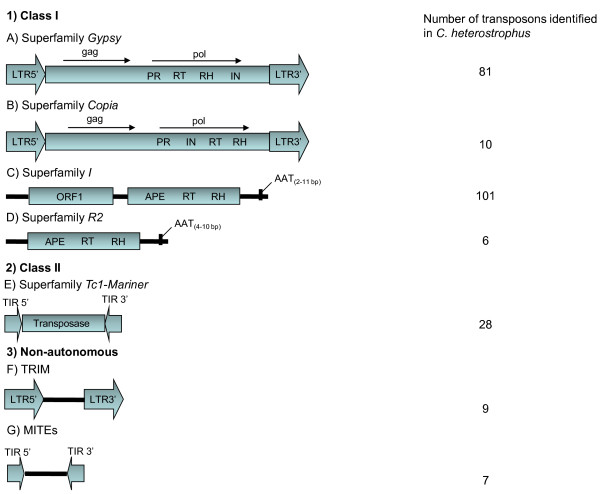
**Basic structure of the major TEs found in the** ***C. heterostrophus*** **race O genome.** In 1, the representatives of class I, superfamily *Gypsy* **(A)**, superfamily *Copia* **(B)**, superfamily *I* **(C)** and superfamily *R2* **(D)**, with their respective coding regions and structural characteristics, are shown. The *pol* region contains the PR (protease), RT (reverse transcriptase), RH (RnaseH) and IN (integrase) domains. ORF2 of superfamily *I* includes APE (apurinic endonuclease), RT (reverse transcriptase) and RH (RNaseH) domains, while TEs of superfamily *R2* have only one ORF with RT (reverse transcriptase) and RH (RNaseH) domains. In 2, elements representative of class II, from the superfamily *Tc1*-*Mariner* **(E)**, are shown. In 3, non-autonomous elements known as TRIMs **(F)** and MITEs **(G)** are shown. LTRs (Long Terminal Repeats) are represented by large arrows and TIRs (Terminal Inverted Repeats) by smaller arrows.

A combination of bioinformatic predictions and manual inspections revealed that 5.9% of the sequenced *C. heterostrophus* race O genome, estimated to be 36 Mb, consists of TEs, of which 61% correspond to complete sequences while the remaining 39% were considered to be degenerate sequences (Table 1). Identified class I elements included LTR retrotransposons belonging to the superfamilies *Copia* and *Gypsy* (Figure 1). A high number of non-LTR retrotransposons belonging to the order LINE, superfamily *I* and superfamily *R2* were also identified (Table 1). Nine non-autonomous elements, annotated as TRIMs, and 66 solo-LTRs were also identified. With regard to class II, only elements pertaining to the order TIR, superfamily *Tc1-Mariner,* were identified (Figure 1) (Table 1). In total, 227 complete element sequences were identified, of which 147 TE sequences exhibited ORFs for major protein domains. These TEs were therefore identified “*in silico*” as being potentially active. Of these potentially active TEs, four elements belonged to the *Copia*, four to the *R2*, eight to the *Tc1-Mariner*, 56 to the *Gypsy* and 68 to the *I* superfamilies (Figure 1). The remaining sequences with identity to TEs were found to be incomplete: they lacked some characteristic structure of the superfamily or no conserved domain was shown and were therefore classified as degenerate sequences (Table 1).

**Table 1:** **Sequences of transposons identified in the genome of** ***C. heterostrophus*** **race O**

Repeat	Class	Order	Superfamily	Number of complete TEs	Number of remaining degenerate copies	Percentage in the genome
**Retrotransposon**	**Class I**	**-**	**-**	**198**	**311**	**5.6**
Non-LTR retrotransposon	Class I	LINE	-	107	80	2.4
Non-LTR retrotransposon	Class I	LINE	*I*	101	80	2.4
Non-LTR retrotransposon	Class I	LINE	*R2*	6	-	<0.00
LTR-retrotransposon	Class I	LTR	-	91	231	3.2
LTR-retrotransposon	Class I	LTR	*Copia*	10	13	0.2
LTR-retrotransposon	Class I	LTR	*Gypsy*	81	143	2.9
TRIM	Class I	LTR	*Copia*	-	7	<0.00
TRIM	Class I	LTR	*Gypsy*	-	2	<0.00
Solo-LTR	Class I	LTR	*Copia*	-	3	<0.00
Solo-LTR	Class I	LTR	*Gypsy*	-	63	<0.00
**DNA transposon**	**Class II**			**29**	**83**	**0.3**
DNA transposon	Class II	TIR	*Tc1-Mariner*	29	76	0.3
MITE	Class II	TIR	*Tc1-Mariner*	-	7	<0.00
**Total Elements**				**227**	**397**	**5.9**

### LTR retrotransposons of the superfamily *Copia*

Ten complete copies of *Copia* superfamily TEs were identified, four of which were potentially active. Two families were identified based on LTR alignments. Analysis of the LTR alignments for these two families against the RepBase database of fungal TEs did not generate any significant results (identity > 80%), demonstrating the existence of two new families of *Copia* elements that are herein termed *Copia-1_CH* and *Copia-2_CH*. Major protein regions with similarity to *Copia*-like elements were found in all of the elements, and are, from 5’ to 3’: a *gag* polypeptide, an integrase domain, a reverse transcriptase domain and an RNase H domain (Figure 1). The family *Copia-1_CH* is composed of nine elements with sizes varying from 5.9 Kb to 6.1 Kb and flanked by 443 bp LTRs. Three of these elements are potentially active and include a single ORF comprising the *gag* and *pol* regions that encodes a 1,525 residue polyprotein. The family *Copia-2_CH* is represented by a single potentially active element. This element is 6,775 bp and is flanked by 1,116 bp LTRs. Additionally, this element has a single ORF comprising the *pol* and *gag* protein domains that encode a 1,525 residue protein. In contrast to the family *Copia-1_CH*, this element recognizes 6 bp insertion site, while the other elements recognize 5 bp sites. The LTRs from all of the elements typically begin with 5’-TG-3’ and end with 5’-CA-3’. Seven TRIM elements and three Solo-LTRs related to elements belonging to the family *Copia-1_CH* were also identified.

### LTR retrotransposons from the superfamily *Gypsy*

A total of 81 complete copies of *Gypsy* superfamily TEs were identified, 56 of which were potentially active. The sizes of the elements varied from 6.7 Kb to 8.4 Kb. Eight TE families belonging to the *Gypsy* superfamily were identified through the alignment of LTRs (Table 2). BLASTN analysis of LTRs against the nucleotide RepBase fungal TE database did not generate any significant results, suggesting that these sequences likely represent eight new TE families, here named *Gypsy-1_CH*, *Gypsy-2_CH, Gypsy-3_CH, Gypsy-4_CH, Gypsy-5_CH, Gypsy-6_CH, Gypsy-7_CH,* and *Gypsy-8_CH*. Each of the potentially active elements has two ORFs related to the *gag* and *pol* regions that vary in size even within the same family (Table 2). Within a given element, the *pol* ORF was encoded by a different reading frame than the *gag* ORF. The *pol* region is composed of the aspartic protease (PR), reverse transcriptase (RT), RNaseH, chromodomain and integrase domains in an organization typical of *Ty3/Gypsy*-like retrotransposons (Figure 1). The family *Gypsy-2_CH* is most frequently represented, with 33 complete elements present in the genome. Unlike the other families, *Gypsy-1_CH* is composed only of potentially active elements, and the *gag* and *pol* ORFs are superimposed, with the first ORF in a +3 reading frame and the second in a +2 reading frame. Similar to *Copia* elements, *Gypsy* elements have LTRs that typically begin with 5’-TG-3’ and end with 5’-CA-3’. Finally, two TRIM elements and 63 Solo-LTRs related to the superfamily *Gypsy* were also identified.

**Table 2:** **TE families belonging to the** ***Gypsy*** **superfamily**

Family	Number of complete TEs	Number of potentially active TEs	Length of TE (Kb)	Length of LTR (bp)	Length gag (aa)	Length ***pol***(aa)
*Gypsy-1_CH*	10	10	6.7	451	645	1,219
*Gypsy-2_CH*	33	24	7.4-7.5	487-516	673-730	1,248-1,471
*Gypsy-3_CH*	11	5	7.5	502-503	717-730	1,379-1,418
*Gypsy-4_CH*	14	11	7.5-8.4	497-518	717-730	1,358-1,420
*Gypsy-5_CH*	5	4	7.4-7.5	485-494	710-730	1,383-1,384
*Gypsy-6_CH*	5	3	7.4	494	717	1,384
*Gypsy-7_CH*	2	0	6.2-7.1	442	-	-
*Gypsy-8_CH*	1	0	6.8	372	-	-

### Non-LTR Retrotransposons

A total of 101 copies of elements belonging to superfamily *I* were identified, 68 of which were considered to be potentially active. These elements varied in size from 5.7 Kb to 6.6 Kb. The alignment of ORF2 between elements identified in the *C. heterostrophus* genome revealed the existence of two groups with identity greater than 80% in at least 80% of the aligned sequences. BLASTN and BLASTP analyses against the RepBase fungi database did not identify similarity greater than 80% with any known sequence, suggesting that these groups represent two new families of non-LTR elements, here termed *I-1_CH* and *I-2_CH*. All the identified elements exhibit two ORFs. ORF1 is similar to the *gag* region, but its role remains unclear. ORF2 contains an apurinic/apyrimidinic endonuclease (APE) domain, a reverse transcriptase (RT) domain and an RNaseH (RH) domain (Figure 1). In the order LINE, this last domain is only present in members of the superfamily *I*. All of the identified elements exhibited tandem repeats of an AAT sequence, with the number of repeats varying between members of the same family (Table 3). Analysis of ORF2 sequences in *I-1_CH* revealed different sized ORFs across the family (Table 3). Six elements belonging to the superfamily *R2* were identified, with three being potentially active and having sizes of 2.2 Kb, 2.7 Kb and 3.2 Kb. The first two elements encode a single 648 residue ORF, while the third encodes a single 879 residue ORF. The ORFs identified for the elements of the superfamily *R2* displayed reverse transcriptase and endonuclease domains.

**Table 3:** **TE families belonging to the** ***I*** **superfamily**

Family	Number of complete TEs	Number of potentially active TEs	Length of TE (Kb)	Length of ORF1 (aa)	Length of ORF2 (aa)	Number of tandem repeats (AAT)
*I-1_CH*	71	49	5.7-5.8	481-513	1,272-1,276	5-11
*I-2_CH*	30	19	5.7	463	1,281	2-6

### Transposable elements of the superfamily *Tc1*-*Mariner*

Twenty-nine complete copies of elements related to the superfamily *Tc1-Mariner* were identified, of which only eight were considered to be potentially active. We decided not to classify these elements according to family because we found a low number of potentially active copies and a high degree of divergence in the structural characteristics of the elements. The size of the complete copies varied between 1.3 Kb and 3.9 Kb. These elements have TIRs varying from 27 bp to 70 bp flanked by a TSD (TA). The elements also have DDE domains characteristic of transposases. The potentially active copies have a single ORF comprising the intact DDE domain, which varied from 436 residues to 610 residues. Seven MITEs elements were also identified. However, RepeatMasker can fail to detect MITEs copies because these sequences are small and lack genes optimizing the identification.

### TEs near genes

The analysis of approximately 5,000 bp of sequence up and down stream of complete TEs identified 76 protein-coding sequences or protein domains near TEs. Several of these genes encode proteins related to the transport of lipids, sugars, nitrogenous bases, and amino acids. Transposons were also found near genes related to the transport of drugs such as the multidrug transporter MFS (Major Facilitator Superfamily) transporters, efflux transporter fnX1 and ABC (ATP Binding Cassette) drug transporter. Other genes associated with important metabolic pathways such as: glycosyl hydrolase, glucosamine-6-phosphate deaminase, asparagine synthetase, chitin synthase, pH-response regulator protein palC, tyrosyl-tRNA synthetase, succinyl-CoA ligase and benomyl/methotrexate resistance protein, among others had TEs nearby (Additional file 1: Table S1).

**Figure 2:**
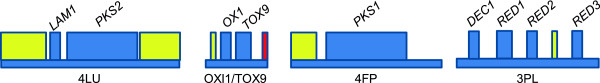
**Architecture of the scaffolds** ***Tox1*** **-associates genes in the race T genome assembly (TMRI).** Tox1 genes are embedded in repeat transposons sequences. Box in yellow and red are sequences related to *Gypsy* and *Copia* retrotransposons, respectively.

Unfortunately, due to the different technologies used to sequence race O strain C5 (Sanger technology) and race T strain C4 (Illumina technology), it was not possible to do a genome-wide comparative analysis of TEs between the two races. The repetitive content returned for genomes sequenced by Illumina tends to underestimate the abundance of repetitive elements because small and repetitive reads are difficult to assemble within long repeat regions using this technology. Thus, the elements identified in race O were not found or were found as small fragments at the ends of the race T scaffolds. However, *C. heterostrophus* race T strain C4 was first sequenced by the Turgeon/Yoder program at the Torrey Mesa Research Institute (TMRI) in 2001. 2× paired-end shotgun sequence coverage was combined with 3× Celera paired end coverage assembled into 300 scaffolds of ~35 Mb. Some *Tox1* scaffolds (597/3 L8 into OXI1/TOX9) were subsequently connected by targeted sequencing. We performed a BLASTN search against the NCBI database (TMRI – *Tox1* sequences) to determine the presence of race O TEs near T-toxin-related virulence genes on scaffolds carrying these genes. The BLASTN analysis against the NCBI database revealed the presence of TEs on scaffolds 4FP (containing the *PKS1* gene), 4 LU (containing *PKS2* and *LAM1* genes), 3PL (containing *DEC1*, *RED1*, *RED2* and *RED3* genes), and OXI1/TOX9 (containing the *OXI1* and *TOX9* genes) (Figure 2).

One partially sequenced retrotransposon element was found 1,748 bp upstream of the *PKS1* gene (GenBank U68040.3). This element was found at the beginning of the scaffold, with a 2,333 bp sequence available. This sequence had 81% identity to a single LTR-Gypsy element identified in race O. One 276 bp LTR fragment was found 921 bp upstream from the *OXI1* gene (GenBank FJ943499.1). That LTR fragment had greater than 80% identity to eight LTR-Gypsy elements identified in race O. Another 226 bp sequence related to the LTRs of Copia elements was identified at the end of the same scaffold. That sequence was found 1,110 bp downstream of the *TOX9* gene (GenBank FJ943499.1). Similarly, that sequence was imperfect and had 88% identity with the sequence of a Copia element identified in the race O genome. A 327 bp sequence similar to other Gypsy LTRs was identified between the *RED2* and *RED3* genes (Genbank AF525909.2), and this sequence showed an identity greater than 85% with 14 LTR sequences from of *Gypsy* elements identified in race O, although it was not possible to detect the complete LTR. A 3,597 bp fragment was identified at the end of the 4 LU scaffold (GenBank DQ186598.2) and was found 52 bp downstream from the *PKS2* gene. Finally, a transposon fragment containing an intact 496 bp LTR was found 107 bp upstream from the *LAM1* gene (GenBank DQ186598.2) (Additional file 2: Figure S1). BLASTN analysis revealed that LTRs in this transposon fragment had more than 80% identity with the 10 *Gypsy-3_CH* family *Gypsy* elements identified in the genome of C*. heterostrophus* race O.

### Evidence of RIP

Evidence of the action of the RIP mechanism was found in elements belonging to the *Tc1*-*Mariner* and *I* superfamilies (Table 4). Evidence of the RIP silencing mechanism was not found in *Copia-1_CH*, *Gypsy-1_CH* or *Gypsy-5_CH* families (Table 4). Interestingly, no elements from these three families were found near genes. In contrast, sequences where RIP was detected were found near coding regions at least once per element.

**Table 4:** TpA/ApT* ratio for transposons in the genome of ***C. heterostrophus***

Superfamily	Family	Number of TEs	Number of TEs near genes	TpA/ApT
***Tc1*** **-** ***Mariner***	-	7	4	1.08
***Tc1*** **-** ***Mariner***	-	4	1	1.04
***Copia***	*Copia-1_CH*	9	0	0.80
***I***	*I-2_CH*	23	4	1.60
***Gypsy***	*Gypsy-1_CH*	10	0	0.85
***Gypsy***	*Gypsy-5_CH*	5	0	0.83

*Standard reference value of the RIP indice is TpA/ApT > 0.89 [60].

**Figure 3:**
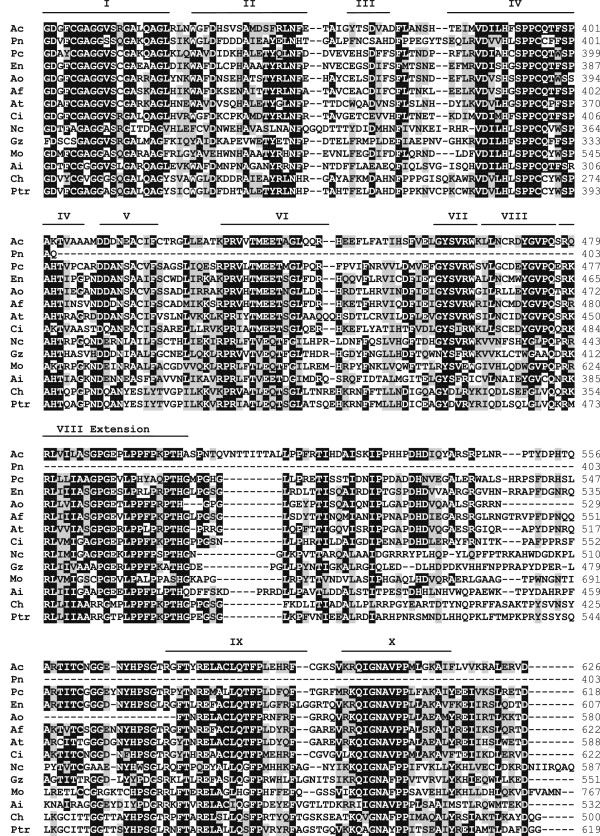
**Predicted proteins and RID (DMT) organization.** The motifs (DMT) are indicated with roman numerals. Ac. *Ajellomyces capsulatus*, Pn. *Phaeosphaeria nodorum*, Pc. *Penicillium chrysogenum* (RID), Ptr. *Pyrenophora tritici-repentis* (DMTA), En. *Emericella nidulans* (DMTA), Ci. *Coccidioides immitis* RS (RID), Ai. *Ascobolus immersus* (Masc1), Ao. *Aspergillus oryzae* (DMTA), Nc. *Neurospora crassa* (RID), Ch *Cochliobolus heterostrophus* (RID), Af. *Aspergillus fumigatus* (DMTA), Gz. *Gibberella zeae* PH-1 (RID), *Aspergillus terreus* (DMTA) and Mo. *Magnaporthe oryzae* (RID).

The alignment between a RID-like sequence found in the *C. heterostrophus* genome database with other sequences already studied in other fungi showed a high degree of similarity. Additionally, the ten conserved domains, proposed by Freitag et al. [25] to be representative of this protein, were found (Figure 3).

### Analysis of ectopic recombination

Transposon sequences whose alignment resulted in nucleotide identity greater than 80% were placed in 15 different groups and evaluated for evidence of ectopic recombination. The groups and the respective numbers of sequences aligned were: Mariner-1 (7), Mariner-2 (4), Mariner-3 (3), I-1 (22), I-2 (23), I-3 (16), I-4 (3), I-5 (25), Gypsy-1 (10), Gypsy-2 (21), Gypsy-3 (10), Gypsy-4 (12), Gypsy-5 (5), Gypsy-6 (3) and Copia-1 (8). Putative ectopic recombination sites were found in all of the transposon superfamilies. The largest number of signs of ectopic recombination was detected in elements of the *Gypsy* superfamily in the group Gypsy-2, belonging to the family *Gypsy-2_CH*, with 35 ectopic recombination events identified by at least four of the recombination methods used (Table 5). In contrast, no evidence of ectopic recombination was found in the groups Mariner-1, Mariner-2, superfamily I-1, superfamily I-4, superfamily I-5, Gypsy-1, Gypsy-7 or Copia-1 (Table 5).

**Table 5:** **Evidence of ectopic recombination events detected in the genome of** ***Cochliobolus heterostrophus***

Groups	Family	Number of sequences utilized	Number of recombination events detected
**Mariner-1**	-	7	0
**Mariner-2**	-	4	0
**Mariner-3**	-	3	1
**I-1**	*I-1_CH*	22	0
**I-2**	*I-1_CH*	23	5
**I-3**	*I-1_CH*	16	2
**I-4**	*I-1_CH*	3	0
**I-5**	*I-2_CH*	25	0
**Gypsy-1**	*Gypsy-1_CH*	10	0
**Gypsy-2**	*Gypsy-2_CH*	21	35
**Gypsy-3**	*Gypsy-3_CH*	10	4
**Gypsy-4**	*Gypsy-4_CH*	12	20
**Gypsy-5**	*Gypsy-5_CH*	5	0
**Gypsy-6**	*Gypsy-6_CH*	3	1
**Copia-1**	*Copia-1_CH*	8	0

### Transcriptional activity of class I and class II transposon sequences

To assess the potential transcriptional activity of the identified TEs, the cluster of TEs generated in support of the *C. heterostrophus* C5 race O genome annotation [5] was analyzed. Of the 88,751 sequences present in the EST cluster, 219 showed significant alignment (E value <10^-5^) with sequences of class I and class II elements. The *I* superfamily was the most frequently represented, with 105 ESTs identified. Ten ESTs related to the *Copia* superfamily, 24 ESTs related to the *Tc1*-*Mariner* superfamily and 80 ESTs related to the *Gypsy* superfamily were also found.

## Discussion

### Transposable elements in the genome of *C. heterostrophus*

Fungi are broadly used in food production, biotechnology and agriculture, in addition to being the primary organisms responsible for the decomposition and recycling of nutrients. However, these microorganisms also cause devastating diseases, primarily in plants, and as such represent an enormous problem for global food security [27]. With the increase in large-scale sequencing projects for fungal genomes, more detailed analyses of the genomes can be performed. These types of analyses have revealed the relevance of the activity of mobile DNA and its role in important genome restructuring events at key moments in evolution [28].

Condon et al. [29] have estimated that 9% of the sequenced genome of *C. heterostrophus* race O strain C5 is composed of repetitive sequences. In the current manuscript, using a combination of bioinformatic predictions and manual adjustments, it was estimated that approximately 6% of these repetitive sequences are TEs, and 12 new families were found. Repetitive sequences generally represent 3 to 20% of the sequenced genomes of fungi; in *Pyrenophora tritici-repentis* [30] and *Setosphaeria turcica* [29] 16% and 12.96%, respectively, of the sequenced genome correspond to repetitive sequences. However, some sequenced genomes such as that of *Ustilago maydis* have low repetitive sequence content, with only 1.1% [31]. In contrast, other fungal genomes with unusual sizes display a large number of repetitive sequences: 85% of the genome of *Blumeria graminis*, estimated at 174 Mb, is represented by repetitive sequences, the largest percentage found to this point in fungi [32].

Approximately 50% of the sequences of TEs identified in the *C. heterostrophus* race O genome belong to the *Gypsy* superfamily of retrotransposons. Retrotransposons are the largest constituent of the repetitive fraction of the genome in phytopathogenic fungi [33]. However, the content of LTR retrotransposons in fungal genomes is highly variable and can range from complete absence, as in *Trichoderma atroviride*, to more than 600 elements, as in *Mycosphaerella graminicola* [19]. Copies of non-autonomous elements from LTR retrotransposons known as TRIMs were also identified. These sequences lack one or more genes essential for transposition. However, non-autonomous elements or defective elements can be cross-activated by similar active elements belonging to different families [17].

Although LTR retrotransposons represent the majority of the TE sequences detected in the genome of *C. heterostrophus* race O, the elements of superfamily *I* were the most abundantly represented in terms of the number of complete and potentially active copies. Non-LTR retrotransposons are the major component found in eukaryotic genomes [34]. However, specifically in fungi, in most species where non-LTR retrotransposons were identified, these are found to be degenerate and comprise no more than 0.5% of the sequenced genome, with the number of copies varying from a single copy in *Botrytis cinerea* to 96 copies in *Chaetomium globosum* [35]. A high percentage (2.4%) of the *C. heterostrophus* genome is related to elements of superfamily *I.* However, the high proportion of non-LTR retrotransposons cannot be easily explained because the abundance and distribution of a particular TE depends on various processes such as limitations on the number of copies imposed by natural selection, which removes deleterious insertions; horizontal and vertical transfer; passive and active inactivation of repeat sequences; and self-regulation of transposition [34, 36–38]. Because the impact of these factors can vary largely, the number of TEs of each species is unique and virtually impossible to predict *a priori* [34]. With regard to LINE elements, elements of the superfamily *R2* were identified that exhibited a single ORF and have a site-specific distribution in the genome. These elements are considered to be more ancestral in the group of non-LTR retrotransposons [17–35].

Copies of elements related to the *Tc1-Mariner* superfamily were also identified. These elements generally encode a single protein known as transposase. Transposases can be divided into various families according to the transposition mechanism. The most representative family is the DD[E/D]-transposase, which contains a characteristic motif of three acidic residues, two of which are aspartic acids and the last is a glutamic acid or, in some cases, a third aspartic acid [39, 40]. All of the potentially active elements identified in this study have the DDE motif. Copies of non-autonomous elements from the *Tc1*-*Mariner* transposons known as MITEs were also identified. These sequences lack one or more genes essential for transposition. However, in various species, a small number of *Tc1*-Mariner elements can be responsible for the origin and activation of a large population of non-autonomous elements [17].

### Elements potentially active

A total of 147 elements, approximately 65% of the complete TEs found, are potentially active. This activity was demonstrated through alignment of the sequences of TEs identified in the *C. heterostrophus* genome against the EST cluster, with the results demonstrating the presence of transcripts related to the major superfamilies of elements identified herein. “In silico” evidence of potential activity has also been reported by Martin et al. [41] in *Laccaria bicolor*. The activation of TEs under stress conditions has been demonstrated in *Aspergillus oryzae* [42] and *Ophiostoma ulmi* [43]. The effect of TE insertion depends on its target locus (exon, intron, promoter, among others), but, in general, the impact of alterations caused by a transposition event is low because deleterious mutations are eliminated. Another source of deleterious effects as a result of TEs is the potential for recombination between elements belonging to the same family. However, one potentially positive effect of the presence of these elements may be that their mutational activity, excluding deleterious insertions, promotes genetic diversity and increases the speed of the adaptation process. In addition, some transposons are linked to genes and control their expression [36, 44, 45].

### Evidence of ectopic recombination

The evolution of chromosomal structure in Dothideomycetes, to a first approximation, appears to be the result of chromosomal rearrangements [5]. In this context, TEs from the same family are considered to be strong sites for ectopic recombination. Ectopic recombination events can influence species adaptation, as they can promote rearrangements (deletion, duplication, inversion or translocation) and chromosomal breaks [46]. In particular, various possible ectopic recombination events involving transposon sequences using the RDP program were detected in the genome of *C. heterostrophus* race O. Therefore, recombination between retrotransposon sequences may have been or is a contributing factor in the reorganization of the *C. heterostrophus* genome. Complex DNA recombination events and the cultivation of monocultures of susceptible maize germplasm containing Tcms are considered to be causes of the Southern Corn Leaf Blight epidemic that occurred in the 1970s with the emergence of the *C. heterostrophus* race T [29].

The presence of non-autonomous elements and solo-LTRs corroborates the notion of the high degree of ectopic recombination between sequences of TEs. These sequences generally result from recombination between sequences of TEs in the genome of *C. heterostrophus* race O. Similarly, the analysis of scaffolds carrying genes responsible for the production of T-toxin in *C. heterostrophus* race T revealed the presence of various solo-LTRs, suggesting a high recombination rate in this region. The *Tox1A* and *Tox1B* loci, related to the production of T-toxin, are associated with a translocation involving race O chromosomes 6 and 12 [10]. The TEs were/are an important ectopic recombination sites and, therefore, can increase variability in this species. Unfortunately, due to the use of Illumina sequencing technology in the genome of race T, further comparisons between the two races could not be conducted, as this type of technology tends to eliminate TEs during assembly of the scaffolds.

### Evidence of RIP

The adaptability of the host may be negatively affected by TEs that can cause gene deletions and duplications, chromosomal rearrangements and alterations in the expression of essential genes. However, some fungi have genetic silencing mechanisms known as RIP mechanisms to control repetitive DNA sequences such as the transposons. Despite evidence of RIP previously reported by Clutterbuck [26] and Ohm et al. [5], no evidence of the presence of the RID protein or the selectivity of the RIP mechanism for euchromatic regions has been reported. In the present study, in addition to the identification of RIP-like mutations in some families of TEs, a RID-like protein, which is known to be an essential part of the RIP machinery in *N. crassa* [25] *,* was identified in the *C. heterostrophus* genome. Another interesting result was that, of all the sequences analyzed, only families of transposons that contain at least one aligned copy near a coding region had RIPed sequences. Elements belonging to the families *Gypsy-1_CH*, *Gypsy-5_CH*, and *Copia-1_CH* did not show strong evidence of RIP activity. No coding sequences were found near any of these transposons, indicating that these TEs are most likely in heterochromatic regions. Thus, RIP in *C. heterostrophus* appears to be a highly selective mechanism, acting only on TE copies inserted near coding regions. A difference in the intensity with which RIP acts between the different transposable elements has been reported in other fungi; in *Stagnospora nodorum,* the *Molly* and *Elsa* transposons are more clearly affected by RIP [47], while in *Aspergillus niger,* RIP is considered a severe event and only two sequences of *AniTa1* elements, of the 15 analyzed, exhibit evidence of RIP. Interestingly, these two sequences are found inserted into ORFs [48]. Another important aspect related to the presence of the RIP silencing mechanism in *C. heterostrophus* has been shown by Ohm et al. [5]. The authors demonstrated that point mutations due to the presence of RIP also occur in regions near TEs. The co-localization of effector genes and TEs, therefore, exposes those genes to high rates of point mutations. This may accelerate the rate of evolution of these genes, providing a potential advantage for the host.

### Transposable elements near coding regions

All genes up to 5,000 bp from the identified TEs were mapped. In addition to the possibility of suffering RIP when at a distance of less than 2,000 bp from the TEs [5], these regions can also be altered in their expression as a result of the presence of TEs. Moreover, rearrangements caused by eventual ectopic recombination can modify gene structure, including toxin biosynthetic locus [49]. Sequences containing genes related to MFS transporters, drug transporters, polyketide synthases and hydrolases were found near TEs. In *Mycosphaerella fijiensis,* where RIP has been shown to be very severe, several MFS and ABC transporters were identified near TEs [50]. Genes related to ABC and MFS transporters have an important role in the transport of drugs and, therefore, provide protection for the organism against toxic products. In plant pathogens, these transporters can be associated with multidrug resistance, virulence and alteration of sensitivity to fungicides [51, 52]. Another group of proteins commonly found near TEs were hydrolases. The two main enzymes found were glycoside hydrolases and lipases. These enzymes are known to play an important role in plant pathogenicity [5]. The fact that TEs are also found near polyketide synthase-encoding genes, major virulence-related genes in race T, is also an indication that these sequences can suffer from the influence of TEs. In *Cochliobolus carbonum*, host-specific toxin genes are situated in transposon-rich regions of the genome [53]. Although the genes listed here are not considered to be essential genes, they play a very important role in the responses to the environment and to the pathogen. TEs have been associated with gene regulation in fungi. For example, in *Aspergillus nidulans* there is evidence of the involvement of transposons in the regulation of gene clusters related to secondary metabolism [54]. In *Pyrenophora tritici-repentis*, another member of the order Pleosporales, the analysis of TEs has suggested the involvement of TEs in the creation of new genes, diversification, horizontal gene transfer and trans-duplication [30]. Additionally, comparative analysis between genomes of pathogenic and non-pathogenic *P. tritici-repentis* isolates showed that pathogenicity in this species emerged through an influx of TEs, which created a genetically flexible situation that enabled an easy response to environmental changes [30].

## Conclusions

In this study, a complete characterization of the major TEs present in the genome of *C. heterostrophus* race O was performed. Twelve new families of transposons were identified, demonstrating the possible role of these elements in the genomic regulation and evolution of *C. heterostrophus*. In *C. heterostrophus,* the RIP silencing mechanism is efficient and selective, allowing movement of elements in heterochromatic regions, but silencing copies that may be inserted into coding regions. The major coding regions influenced by RIP mechanism were also characterized. Additionally, it was shown that ectopic recombination promoted by TEs appears to be the major event in the genome reorganization of this species and that a large number of elements are still potentially active.

## Methods

### Identification and classification of transposable elements

The genome of *C. heterostrophus* race O strain C5 v2.0 was obtained from the Joint Genome Institute (JGI) database (http://www.jgi.doe.gov/genome-projects/). Identification and classification of TE sequences in the genome of *C. heterostrophus* were performed using the RepeatMasker program (A.F.A. Smit, R. Hubley & P. Green RepeatMasker at http://repeatmasker.org). This program identifies copies of TEs by comparing genome sequences with sequences present in a previously described library of TEs (RepBase 16.12: http://www.girinst.org/repbase/update/index.html) [55]. In this work, the library of fungal TE sequences (fngrep.ref) was used. The following parameters were used for this search: “RM_BLAST” as the search model; “slow search” to obtain a search 0-5% more sensitive than the standard search; “fungi” to specify the species or group of input sequences; and “alignment” to generate an output file showing the alignment. However, this program only marks genome regions having identity with database TE sequences, and in many cases it is not possible to determine element boundaries. For this reason, the identification of class I LTRs was performed using the LTR-Finder program (http://tlife.fudan.edu.cn/ltr_finder/) [56] and the Repeat Finder program [57]. Class II TIRs were identified using the Repeat Finder program [57]. Complete non-LTR transposons were identified by the structural characteristics of each superfamily, including the number of ORFs, duplicate sites and the presence of repetitive regions at the 3’ end. Analysis of ORFs within TE coding regions was performed using the Expasy (http://expasy.org/) and Orf-finder (http://www.ncbi.nlm.nih.gov/projects/gorf/) programs. TE insertion sites, or TSRs (Target Site Repeat), were characterized by direct search of the sequences that flanked each TE.

Sequences identified in this way were classified as complete elements, potentially active elements or degenerate sequences. Complete elements were those sequences with similarity to proteins related to the transposition machinery, conserved terminal repeats and target site duplications (TSDs), but lacking intact ORFs. Potentially active elements were complete elements that exhibited intact protein domains and ORFs characteristic of the superfamily of transposons. Finally, degenerate sequences were those sequences that displayed identity with consensus sequences of the major characterized TEs (RepBase). However, degenerate sequences lacked structural characteristics or sequences encoding transposition-related proteins.

To define families, the classification system proposed by Wicker et al. [17] was used. In this system, families are defined as groups of TEs with more than 80% identity between coding regions, internal domains or terminal repeat regions in at least 80% of the aligned sequences. For this definition, the ORF2 coding region was used to classify non-LTR elements. We chose to use ORF2 coding regions to classify these elements at the family level because non-LTR elements do not have terminal repeat regions and their 5’-untranslated regions (UTRs) and 3’UTRs are highly variable. LTR sequences were used to classify *Copia* and *Gypsy* retrotransposons because according to Wicker et al. [17] the terminal repeat regions are the most rapidly evolving portion the elements and therefore they provide greater specificity for the definition of families than do protein-coding regions. To determine the existence of new TEs families, elements from each family were analyzed by BLAST against the database of fungal TEs (fngrep.ref) deposited in RepBase (http://www.girinst.org/repbase/update/index.html) [55]. Finally, the elements were named according to the nomenclature of Kapitonov and Jurka [58], and representative TE sequences from novel families were submitted to the database at http://www.girinst.org/repbase/update/browse.php with the following identifiers: *Copia-1_CH* and *Copia-2_CH* (Superfamily *Copia*); *Gypsy-1_CH*, *Gypsy-2_CH, Gypsy-3_CH, Gypsy-4_CH, Gypsy-5_CH, Gypsy-6_CH, Gypsy-7_CH,* and *Gypsy-8_CH* (Superfamily *Gypsy*); *I-1_CH* and *I-2_CH* (Superfamily *I*).

### Transposable elements near coding regions

After searching for complete TEs, approximately 5,000 bp upstream and downstream of each TE were analyzed by BLASTX (http://www.ncbi.nlm.nih.gov/BLAST) against the RefSeq_protein database (Reference Sequence Protein) to determine the existence of protein-coding sequences near the TEs. The threshold used for protein identification was E-value < 10^-20^ and identity > 50%.

A BLASTN search was performed against the NCBI database to determine the presence of race O TEs near T-toxin-related virulence genes previously identified in race T sequence scaffolds. The sequences analyzed were: scaffold 4FP, carrying the *PKS1* gene [9, 12]; scaffold 4 LU, carrying the *PKS2* and *LAM1* genes [11, 12]; scaffold 3PL, carrying the *DEC1*, *RED1*, *RED2* and *RED3* genes [12, 13]; and scaffold OXI1/TOX9, carrying the *TOX9* and *OXI1* genes [12]. The sequences of scaffolds 4FP, 4 LU, 3PL, and OXI1/TOX9 can be accessed in GenBank using the accession numbers U68040.3, DQ186598.2, AF525909.2 and FJ943499.1, respectively.

### Evidence of RIP

DNA sequences associated with the synthesis of transposition proteins were analyzed for dinucleotide frequency and RIP index. Regions comprising *gag* and *pol* regions (*Copia*), *pol* region (*Gypsy*), transposase (*Tc1*-*Mariner*) and ORF2 (*I*) were aligned using the Mega 4 program [59]. Only alignments containing pairs of sequences from the same family that had 100% coverage and an identity greater than 80% but lacked evidence of ectopic recombination were aligned and later used in the RipCal program [60] to calculate TpA/ApT index. The TpA/ApT index is a simple index that measures the frequency of RIP products (TpA) with a false positive relation due to ApT-rich regions. High TpA/ApT values indicate a strong response to RIP [60].

A search for the RID protein (DNA methyltransferase – DMT) was performed in the JGI *C. heterostrophus* genome database using the keyword “methyltransferase”. Alignments of different DMTs were performed to demonstrate the presence of conserved domains. GenBank accession numbers for C5-cytosine methyltransferase genes are as follows: *Ajellomyces capsulatus* (XP_001539629), *Phaeosphaeria nodorum* (XP_001797905), *Penicillium chrysogenum* (CAP86663), *Pyrenophora tritici-repentis* (XP_001935966), *Emericella nidulans* (AF428247), *Coccidioides immitis* RS (XP_001239116), *Ascobolus immersus* (AF025475), *Aspergillus oryzae* (BAE61916), *Neurospora crassa* (AAM27408), *Cochliobolus heterostrophus* (Scaffold 2, starting at 180,615 bp and ending at 182,404 bp), *Aspergillus fumigatus* (XP_747703), *Gibberella zeae* PH-1 (XP_388824), *Aspergillus terreus* (XP_001209776), and *Magnaporthe oryzae* (XP_366719).

### Evidence of ectopic recombination

Evidence of ectopic recombination in the transposon sequences found in the *C. heterostrophus* genome was analyzed using RDP (recombination Detection Program) [61], Geneconv [62], Bootscan [63], Maximum Chi Square [64], Chimaera [65], Sister Scan [66] and 3Seq [67] implemented in RDP, version 3.0 [68]. TEs belonging to each family were aligned using the Mega 4 program [59], and sequence pairs with less than 80% identity were discarded. Alignments were analyzed using standard configurations for the different methods with a 0.005 cutoff for the Bonferroni-corrected p-value. Only ectopic recombination events detected by at least four of the methods used were considered to be reliable.

### Transcribed sequences of class I and class II TEs

In order to assess potential TE transcriptional activity, the EST-cluster database [5] was inspected for sequences corresponding to the various TEs described herein. The EST-cluster database was previously constructed specifically to support *C. heterostrophus* C5 race O genome annotation. The library was obtained from the JGI genome database (http://www.jgi.doe.gov/). Sequences of complete elements and potentially active elements identified were aligned by BLASTN against a total of 88,751 expressed sequence tags (ESTs) [5]. ESTs from TEs with significant BLAST scores (E value < 10^-5^) in relation to the elements identified here were considered.

### Availability of supporting data

The genomes and EST-cluster of *C. heterostrophus* were downloaded from the Joint Genome Institute (JGI) database (http://www.jgi.doe.gov/genome-projects/). TE sequences from novel families are deposited at http://www.girinst.org/repbase/update/browse.php with the following identifiers: *Copia-1_CH* and *Copia-2_CH* (Superfamily *Copia*); *Gypsy-1_CH*, *Gypsy-2_CH, Gypsy-3_CH, Gypsy-4_CH, Gypsy-5_CH, Gypsy-6_CH, Gypsy-7_CH,* and *Gypsy-8_CH* (Superfamily *Gypsy*); *I-1_CH* and *I-2_CH* (Superfamily *I*). Supporting data are included as additional files.

## Electronic supplementary material

Additional file 1: Table S1: Sequences coding proteins downstream and upstream of full copies of the transposable elements. The table contains an analysis of the regions approximately 5,000 bp upstream and downstream of each transposable element. (DOCX 41 KB)

Additional file 2: Figure S1: Transposon fragment containing an intact LTR found upstream from the LAM1 gene in the genome of *C. heterostrophus* race T. The figure contains the Scaffold 4 LU with transposons and Tox sequences. (DOCX 29 KB)
