# Supplementary material for: Characterization and potential evolutionary impact of transposable elements in the genome of Cochliobolus heterostrophus
Source: BMC Genomics. 2014 Jun 28;15(1):536. doi: 10.1186/1471-2164-15-536 (PMC4112212; doi:10.1186/1471-2164-15-536)
Supplement: Supplementary file 1 — Additional file 1: Table S1: Sequences coding proteins downstream and upstream of full copies of the transposable elements. The table contains an analysis of the regions approximately 5,000 bp upstream and downstream of each transposable element. (DOCX 41 KB) [file 12864_2014_6280_MOESM1_ESM.docx]

**Table 1** **Sequences coding proteins downstream and upstream of full copies of the transposable elements**

| **Scaffold** | **Superfamily-**  **Identification** | **Gene** | **Approximate**  **distance (pb)** | **Sequence identity (%)** | **Sequence similarity (%)** | **Access Genbank** |
| --- | --- | --- | --- | --- | --- | --- |
| 55 | *I-2* | scramblase family protein  tRNA-splicing endonuclease subunit Sen2  5'-3' exoribonuclease | U: 3,248  U: 1,049  D: 4,625 | 79  65  88 | 85  77  93 | [XP_001941325.1](http://www.ncbi.nlm.nih.gov/protein/189209986?report=genbank&log$=protalign&blast_rank=2&RID=8FNE7DC001R)  [XP_003845125.1](http://www.ncbi.nlm.nih.gov/protein/396498051?report=genbank&log$=protalign&blast_rank=4&RID=8FNE7DC001R)  [XP_003845052.1](http://www.ncbi.nlm.nih.gov/protein/396497754?report=genbank&log$=protalign&blast_rank=2&RID=8FNGYEV601R) |
| 21 | *I-19* | beta-glucosidase 1 | D: 1,950 | 59 | 72 | [XP_003711559.1](http://www.ncbi.nlm.nih.gov/protein/389627812?report=genbank&log$=protalign&blast_rank=1&RID=8XAJJ8J501R) |
| 17 | *Gypsy-25* | phosphoribosylformylglycinamidine synthase | U: 1,579 | 92 | 96 | [XP_001941251.1](http://www.ncbi.nlm.nih.gov/protein/189209838?report=genbank&log$=protalign&blast_rank=1&RID=8ZZHE050014) |
| 17 | *I-28* | glycosyl hydrolase, family 92 protein | U: 4,606 | 56 | 69 | [XP_001818243.2](http://www.ncbi.nlm.nih.gov/protein/317140529?report=genbank&log$=protalign&blast_rank=1&RID=905RP1T501R) |
| 16 | *Tc1-Mariner-5* | MFS multidrug transporter | U: 3,832 | 56 | 75 | [XP_003233701.1](http://www.ncbi.nlm.nih.gov/protein/327298015?report=genbank&log$=protalign&blast_rank=5&RID=90VJ286Y01R) |
| 16 | *I-29* | multidrug resistance protein fnx1 | U: 1,808 | 94 | 97 | [XP_001942159.1](http://www.ncbi.nlm.nih.gov/protein/189211660?report=genbank&log$=protalign&blast_rank=2&RID=925WHANA014) |
| 15 | *Tc1-Mariner-10* | ubiquitin-conjugating enzyme E2 | U: 1,571 | 66 | 67 | [XP_003841380.1](http://www.ncbi.nlm.nih.gov/protein/396482026?report=genbank&log$=protalign&blast_rank=4&RID=929XYVM901R) |
| 15 | *I-30/31* | P450 monooxygenase  aldehyde dehydrogenase-like protein, | U: 1,070  D: 207 | 51    65 | 70  79 | [XP_003857436.1](http://www.ncbi.nlm.nih.gov/protein/398412218?report=genbank&log$=protalign&blast_rank=16&RID=92GA6AAE014)  [XP_003856215.1](http://www.ncbi.nlm.nih.gov/protein/398409500?report=genbank&log$=protalign&blast_rank=5&RID=92GC9N8D016) |
| 14 | *I-39* | plasma membrane ATPase    FAD dependent oxidoreductase | U: 4,228  D: 3,431 | 94  66 | 95  78 | [XP_001933819.1](http://www.ncbi.nlm.nih.gov/protein/189194962?report=genbank&log$=protalign&blast_rank=2&RID=930681H3014)  [XP_003840292.1](http://www.ncbi.nlm.nih.gov/protein/396477535?report=genbank&log$=protalign&blast_rank=38&RID=930CJNNK01R) |
| 14 | *R2-1* | urea amidolyase, | U: 3,910 | 66 | 78 | [XP_002150044.1](http://www.ncbi.nlm.nih.gov/protein/212539778?report=genbank&log$=protalign&blast_rank=1&RID=9312J2N201R) |
| 13 | *I-40* | L-ascorbate oxidase  aldehyde dehydrogenase | U: 1,045  U: 4,175 | 51  51 | 66  64 | [XP_003713236.1](http://www.ncbi.nlm.nih.gov/protein/389631166?report=genbank&log$=protalign&blast_rank=2&RID=960Z3VV501R)  [XP_002149594.1](http://www.ncbi.nlm.nih.gov/protein/212538877?report=genbank&log$=protalign&blast_rank=20&RID=960Z3VV501R) |
| 12 | *Tc1-Mariner-11* | AMP dependent CoA ligase, putative  HypA | D: 1,500  D: 2,971 | 52  58 | 69  76 | [XP_002144856.1](http://www.ncbi.nlm.nih.gov/protein/212529398?report=genbank&log$=protalign&blast_rank=3&RID=97ENWM8A01R)  [XP_002844733.1](http://www.ncbi.nlm.nih.gov/protein/296808789?report=genbank&log$=protalign&blast_rank=4&RID=97ENWM8A01R) |
| 12 | *Tc1-Mariner-12* | cellulose-binding protein  ser/Thr protein phosphatase family protein  lactonohydrolase | U: 4,866  D: 833  D: 3,148 | 72  85  68 | 84  94  83 | [XP_003834860.1](http://www.ncbi.nlm.nih.gov/protein/396460496?report=genbank&log$=protalign&blast_rank=4&RID=97G9RMVC014)  [XP_001940921.1](http://www.ncbi.nlm.nih.gov/protein/189209177?report=genbank&log$=protalign&blast_rank=1&RID=97GCWZ5V01R)  [XP_001941027.1](http://www.ncbi.nlm.nih.gov/protein/189209389?report=genbank&log$=protalign&blast_rank=2&RID=97GCWZ5V01R) |
| 12 | *Gypsy-32* | cyclopentanone 1,2-monooxygenase | U: 3,883 | 57 | 72 | [XP_001942142.1](http://www.ncbi.nlm.nih.gov/protein/189211625?report=genbank&log$=protalign&blast_rank=30&RID=97MFVFZE01R) |
| 11 | *I-47* | DUF895 domain membrane protein  glucosamine-6-phosphate deaminase  UNC93-like protein | D: 194  D: 3,914  D: 1,324 | 84  93  51 | 93  96  69 | [XP_003843322.1](http://www.ncbi.nlm.nih.gov/protein/396490376?report=genbank&log$=protalign&blast_rank=7&RID=9FAEHH6X016)  [XP_001938488.1](http://www.ncbi.nlm.nih.gov/protein/189204306?report=genbank&log$=protalign&blast_rank=9&RID=9FAEHH6X016)  [XP_001826644.1](http://www.ncbi.nlm.nih.gov/protein/169784366?report=genbank&log$=protalign&blast_rank=53&RID=9FAEHH6X016) |
| 11 | *I-48* | ankyrin repeat-containing protein, putative | D: 2,486 | 63 | 77 | [XP_002376776.1](http://www.ncbi.nlm.nih.gov/protein/238491078?report=genbank&log$=protalign&blast_rank=1&RID=9FD9G19M016) |
| 10 | *I-50* | hybrid polyketide synthetase/nonribosomal peptide synthetase, | U: 153 | 77 | 86 | [XP_003849178.1](http://www.ncbi.nlm.nih.gov/protein/398391436?report=genbank&log$=protalign&blast_rank=1&RID=9G1V3570014) |
| 9 | *Tc1-Mariner-17* | FAD binding domain containing protein  histidinol dehydrogenase | D: 411  D: 3,773 | 81    69 | 90  79 | [XP_001942357.1](http://www.ncbi.nlm.nih.gov/protein/189212061?report=genbank&log$=protalign&blast_rank=2&RID=9G67YBXU01R)  [XP_001263973.1](http://www.ncbi.nlm.nih.gov/protein/119494107?report=genbank&log$=protalign&blast_rank=27&RID=9G67YBXU01R) |
| 8 | *Gypsy-40* | 3-ketoacyl-CoA thiolase  succinyl-CoA ligase subunit beta | U: 2,918  U: 2,477 | 85    72 | 90  78 | [XP_003834539.1](http://www.ncbi.nlm.nih.gov/protein/396459853?report=genbank&log$=protalign&blast_rank=4&RID=9HZCU8C4016)  [XP_003834541.1](http://www.ncbi.nlm.nih.gov/protein/396459857?report=genbank&log$=protalign&blast_rank=6&RID=9HZCU8C4016) |
| 07 | *I-58* | endoglucanase IV precursor | U: 1,968 | 76 | 85 | [XP_001931371.1](http://www.ncbi.nlm.nih.gov/protein/189190064?report=genbank&log$=protalign&blast_rank=3&RID=9J7EAAGG01R) |
| 07 | *I-59* | amidohydrolase 2 | D: 1,236 | 51 | 65 | [XP_003840511.1](http://www.ncbi.nlm.nih.gov/protein/396478342?report=genbank&log$=protalign&blast_rank=11&RID=9J8SCFPX01R) |
| 7 | *R2-2* | ABC drug exporter AtrF | U: 1,027 | 61 | 75 | [XP_001396441.1](http://www.ncbi.nlm.nih.gov/protein/145248385?report=genbank&log$=protalign&blast_rank=2&RID=9JM9RRB901R) |
| 7 | *I-61* | scramblase family protein  tRNA-splicing endonuclease subunit Sen2  5'-3' exoribonuclease 2  asparagine synthetase | U: 2,661  U: 462  D: 3,649  D: 1 | 79  65  91  88 | 85  77  94  93 | [XP_001941325.1](http://www.ncbi.nlm.nih.gov/protein/189209986?report=genbank&log$=protalign&blast_rank=2&RID=9JR7KK8401R)  [XP_003845125.1](http://www.ncbi.nlm.nih.gov/protein/396498051?report=genbank&log$=protalign&blast_rank=4&RID=9JR7KK8401R)  [XP_001941197.1](http://www.ncbi.nlm.nih.gov/protein/189209730?report=genbank&log$=protalign&blast_rank=1&RID=9JR82MPP01R)    [XP_001941322.1](http://www.ncbi.nlm.nih.gov/protein/189209980?report=genbank&log$=protalign&blast_rank=76&RID=9JR82MPP01R) |
| 7 | *I-63* | chitin synthase  vi polysaccharide biosynthesis protein vipA/tviB  benomyl/methotrexate resistance protein | U: 2,597  U: 603  D: 2,954 | 50  84    86 | 65  91  92 | [XP_001933885.1](http://www.ncbi.nlm.nih.gov/protein/189195094?report=genbank&log$=protalign&blast_rank=5&RID=9K5GSW98014)  [XP_001933886.1](http://www.ncbi.nlm.nih.gov/protein/189195096?report=genbank&log$=protalign&blast_rank=9&RID=9K5GSW98014)  [XP_001931654.1](http://www.ncbi.nlm.nih.gov/protein/189190630?report=genbank&log$=protalign&blast_rank=3&RID=9K5RNB74014) |
| 6 | *Gypsy-45* | trichothecene 3-O-acetyltransferase | U: 1,158 | 75 | 85 | [XP_001930722.1](http://www.ncbi.nlm.nih.gov/protein/189188766?report=genbank&log$=protalign&blast_rank=2&RID=9MJUKNV9016) |
| 6 | *I-64* | fumarylacetoacetate hydrolase family protein  gentisate 1,2-dioxygenase  salicylate hydroxylase | U: 3,082  D: 74  D: 2,349 | 75  60  53 | 86  72  66 | [XP_002372444.1](http://www.ncbi.nlm.nih.gov/protein/238482411?report=genbank&log$=protalign&blast_rank=4&RID=9MM87MUU016)  [XP_001398569.2](http://www.ncbi.nlm.nih.gov/protein/317037499?report=genbank&log$=protalign&blast_rank=4&RID=9MM9J42N014)  [XP_001817373.2](http://www.ncbi.nlm.nih.gov/protein/317139256?report=genbank&log$=protalign&blast_rank=16&RID=9MM9J42N014) |
| 5 | *Gypsy-48* | Membrane proteinTapt1/CMV receptor | U: 1,651 | 53 | 67 | [EKG12821.1](http://www.ncbi.nlm.nih.gov/protein/407919591?report=genbank&log$=protalign&blast_rank=4&RID=9N4UNXKP016) |
| 5 | *Tc1-Mariner-21* | ABC drug exporter AbcA | D: 1,218 | 68 | 82 | [XP_001276075.1](http://www.ncbi.nlm.nih.gov/protein/121717324?report=genbank&log$=protalign&blast_rank=8&RID=9N58A3V301R) |
| 5 | *R2-3* | Aha1domain family protein  MFS transporter | U: 4,000  U: 1,401 | 78  51 | 83  68 | [XP_001932744.1](http://www.ncbi.nlm.nih.gov/protein/189192811?report=genbank&log$=protalign&blast_rank=6&RID=9N6617FP01R)  [XP_753036.1](http://www.ncbi.nlm.nih.gov/protein/70996562?report=genbank&log$=protalign&blast_rank=36&RID=9N6617FP01R) |
| 5 | *Gypsy-51* | MFS transporter | D: 4,643 | 58 | 72 | [XP_751264.2](http://www.ncbi.nlm.nih.gov/protein/146324544?report=genbank&log$=protalign&blast_rank=9&RID=9N9KE8XN01R) |
| 5 | *Gypsy-52* | NAD-dependent epimerase/dehydratase | U: 4,619 | 62 | 75 | [XP_003000612.1](http://www.ncbi.nlm.nih.gov/protein/302405551?report=genbank&log$=protalign&blast_rank=27&RID=9NAJSY00014) |
| 5 | *Gypsy-53* | glycosyl hydrolase | D: 3,014 | 59 | 71 | [XP_003000615.1](http://www.ncbi.nlm.nih.gov/protein/302405557?report=genbank&log$=protalign&blast_rank=17&RID=9NBTRF0S01R) |
| 5 | *Gypsy-55* | prenyltransferase, UbiA family protein | D: 3,620 | 62 | 75 | [XP_001274298.1](http://www.ncbi.nlm.nih.gov/protein/121713374?report=genbank&log$=protalign&blast_rank=6&RID=9RPDCCK901R) |
| 4 | *R2-4* | purine permease  5-methylthioadenosine/S-adenosylhomocysteine deaminase n1 | D: 1,260  D: 3,541 | 88%  78 | 96  88 | [XP_001938368.1](http://www.ncbi.nlm.nih.gov/protein/189204065?report=genbank&log$=protalign&blast_rank=3&RID=A1P5BTPR014)  [XP_001938369.1](http://www.ncbi.nlm.nih.gov/protein/189204067?report=genbank&log$=protalign&blast_rank=4&RID=A1P5BTPR014) |
| 4 | *Tc1-Mariner-22* | methylmalonate-semialdehyde dehydrogenase  calreticulin precursor | U: 3,462  U: 1,293 | 89  81 | 93  86 | [XP_001938331.1](http://www.ncbi.nlm.nih.gov/protein/189203991?report=genbank&log$=protalign&blast_rank=1&RID=A1RDRYMR014)  [XP_001938332.1](http://www.ncbi.nlm.nih.gov/protein/189203993?report=genbank&log$=protalign&blast_rank=38&RID=A1RDRYMR014) |
| 4 | *Gypsy-56* | multidrug resistance protein fnx1  pH-response regulator protein palC  amino acid permease | U: 4,077  U: 907  D: 3,101 | 70  86  63 | 78  92  78 | [XP_001938334.1](http://www.ncbi.nlm.nih.gov/protein/189203997?report=genbank&log$=protalign&blast_rank=5&RID=A1REAJCA014)  [XP_001938336.1](http://www.ncbi.nlm.nih.gov/protein/189204001?report=genbank&log$=protalign&blast_rank=2&RID=A1REAJCA014)  [XP_002481020.1](http://www.ncbi.nlm.nih.gov/protein/242787498?report=genbank&log$=protalign&blast_rank=6&RID=A1UC4U8J014) |
| 4 | *Gypsy-59* | FAD binding domain protein | D: 1,567 | 54 | 68 | [XP_001266570.1](http://www.ncbi.nlm.nih.gov/protein/119499626?report=genbank&log$=protalign&blast_rank=5&RID=A1ZK1M2W014) |
| 3 | *I-70* | SacI domain containing protein  class II aldolase/adducin domain containing protein | U: 1,747  D: 233 | 82  71 | 91  78 | [XP_001940981.1](http://www.ncbi.nlm.nih.gov/protein/189209297?report=genbank&log$=protalign&blast_rank=1&RID=A25W8F4R01R)    [XP_001940875.1](http://www.ncbi.nlm.nih.gov/protein/189209085?report=genbank&log$=protalign&blast_rank=4&RID=A25VFY5P01R) |
| 3 | *Tad-73* | amino-acid permease | D: 4,939 | 57 | 76 | [XP_003666115.1](http://www.ncbi.nlm.nih.gov/protein/367033665?report=genbank&log$=protalign&blast_rank=8&RID=A2AVUTCU016) |
| 3 | *Gypsy-62* | RNA-directed RNA polymerase 2  hingosine hydroxylase | U: 3,531  D: 4,445 | 53  82 | 67  92 | [XP_001940806.1](http://www.ncbi.nlm.nih.gov/protein/189208947?report=genbank&log$=protalign&blast_rank=2&RID=A2C9T02T01R)  [XP_001940804.1](http://www.ncbi.nlm.nih.gov/protein/189208943?report=genbank&log$=protalign&blast_rank=2&RID=A2CANFTB01R) |
| 3 | *I-74* | tyrosyl-tRNA synthetase | D: 761 | 94 | 97 | [XP_001932336.1](http://www.ncbi.nlm.nih.gov/protein/189191994?report=genbank&log$=protalign&blast_rank=30&RID=A2EPER6T016) |
| 2 | *Gypsy-69* | major myo-inositol transporter iolT (sugar transporter)  dipeptidyl peptidase 4 | U: 4,761  D: 4,403 | 84  60 | 92  77 | [XP_001933447.1](http://www.ncbi.nlm.nih.gov/protein/189194217?report=genbank&log$=protalign&blast_rank=1&RID=A47KPSEH016)  [XP_003711833.1](http://www.ncbi.nlm.nih.gov/protein/389628360?report=genbank&log$=protalign&blast_rank=6&RID=A47MN41W01R) |
| 2 | *I-81* | FAD binding domain containing protein | U: 4,346 | 75 | 86 | [XP_001939593.1](http://www.ncbi.nlm.nih.gov/protein/189206518?report=genbank&log$=protalign&blast_rank=2&RID=A4EMGY5X01R) |
| 2 | *Tc1-Mariner-26* | phospholipase, patatin family protein | D: 2,985 | 68 | 82 | [XP_001727333.2](http://www.ncbi.nlm.nih.gov/protein/317136856?report=genbank&log$=protalign&blast_rank=2&RID=A4G3K2BV01R) |
| 1 | *Gypsy-73* | vegetative incompatibility protein HET-E-1 | D: 4,185 | 62 | 73 | [XP_001938086.1](http://www.ncbi.nlm.nih.gov/protein/189203501?report=genbank&log$=protalign&blast_rank=8&RID=A4HBC63K01R) |
| 1 | *Gypsy-74* | isoflavone reductase family protein | U: 1,338 | 84 | 94 | [XP_001934235.1](http://www.ncbi.nlm.nih.gov/protein/189195794?report=genbank&log$=protalign&blast_rank=6&RID=A4JZ4VVP016) |
| 1 | *I-86* | chromatin structure-remodeling complex protein rsc1 | D: 4,664 | 85 | 90 | [XP_001936787.1](http://www.ncbi.nlm.nih.gov/protein/189200901?report=genbank&log$=protalign&blast_rank=2&RID=A4RFHZVY014) |
| 1 | *I-86B* | kinesin light chain 3 | D: 1 | 68 | 79 | [XP_001941711.1](http://www.ncbi.nlm.nih.gov/protein/189210760?report=genbank&log$=protalign&blast_rank=1&RID=A4Z0A7HS014) |
| 1 | *I-89* | glycoside hydrolase family 93 protein | U: 2,047 | 57 | 69 | [XP_003665690.1](http://www.ncbi.nlm.nih.gov/protein/367032814?report=genbank&log$=protalign&blast_rank=6&RID=A50SGUKK01R) |
| 1 | *I-92/93* | esterase/lipase  esterase/lipase | D: 4,653  U: 2,637 | 62  62 | 81  81 | [XP_001939581.1](http://www.ncbi.nlm.nih.gov/protein/189206493?report=genbank&log$=protalign&blast_rank=25&RID=A5JJVUXP01R)  [XP_001939581.1](http://www.ncbi.nlm.nih.gov/protein/189206493?report=genbank&log$=protalign&blast_rank=25&RID=A5JJVUXP01R) |

U: Upstream

D: Downstream
