# Supplementary material for: Characterization and potential evolutionary impact of transposable elements in the genome of Cochliobolus heterostrophus
Source: BMC Genomics. 2014 Jun 28;15(1):536. doi: 10.1186/1471-2164-15-536 (PMC4112212; doi:10.1186/1471-2164-15-536)
Supplement: Supplementary file 2 — Additional file 2: Figure S1: Transposon fragment containing an intact LTR found upstream from the LAM1 gene in the genome of C. heterostrophus race T. The figure contains the Scaffold 4 LU with transposons and Tox sequences. (DOCX 29 KB) [file 12864_2014_6280_MOESM2_ESM.docx]

TAATATCTTAGATATTATTCTAATATCTAATTTTATTATTCTATTTAGCTATTCGTTTATAGGTAGTAAG

TAGAACTTAGTACTCTTTTTAAGTTAAGGTAATAAAGAAAGGTTTTCTAATAAGTAGAGTTTATTAGTAG

TTCTCTATCTAATATTATTTATTATAAGACGCTATATAGTTAAATAACTTCGCGTAACTAGGCCTAGGCT

AGATTAACTCTATTCTAATTAGTGTAAGCTAGTACGTAATAGGCTATTTTAGTTAATTTATATATAATTA

CTAATATTAAGTTATAAACTATATTAGATAGCTTACTTAGTAGAAGTTCTATAATAAAATCTATAGTTAT

ATTCTCTTATAGTTTATTAAGTAAAGGTATTAAAGTAAGTAGTTTATAAGAAGCGTATTTTCTTAAAGTT

ACTTTCTAGTAAACTAGATAAGATTTATAGTACTCTTAAATATTCTTTCTTATTCTCTTCTACTTATATT

AACGCGATAGCTTCTTTAATGTACGCTTTATACTTATATAGCCTAAAAGTAGATTATTATAATATTCTTT

AAGTGCCTTTACTTTATTCTATAATAATATAGCCTTTTCCTTAGTACTTTTAGCTTATTTTAAGCTAATT

TAAGGTAATATTATCTCTACTTCTCTTTTTTATAGTAGATACGTCTATTAACTACACTTCTATCTTAGTT

TTATTCTTATAAAATCTAGAGTCTTTATTATTTATAGTATTCTATCTTAAAAACCTTTTATATACTAGCT

TTAGTTTCTCTCTTTTAGTTACTTTAGTTTTACTTCTAGTTATAAAGTTTAATTATAAGAGATCTAACTT

AAGTATATTTCTTATAGCTTCTATAAATTCTTAGTTCTATTTTTAAGTTAGAGATTTAGCTAACTCTATA

TAGTTAGGTTAGTAAAAAGAGCTATTTACTAGGTTTATTTAGTCTAGTTAGTATAGAATAGTAAAATTAT

ACTTAGATAGAGCTAGATAGAACTAAACTTACCTATTATTAAGCGTTTTAGTCTCTATAAACTATTTAAA

GTTATTATAATCTATAATTAGCTTAAAATAAATTCTCTTAAGATATCTCTACTAGTATTAGAGGCTTACT

ACTATTACTAGTAGCTCTTAGTTATAAATATTATAGTTATACTTAGTAGTAATTAACTTATAAGAGTAGA

AGTTTACTAGTCTTTAATAATTATCTAAATATTAGTTAAGAATTCTTACTATTACTCTTCTTAAAGCGTC

TACTTATACTTAGGTCTTTTAATTTAGATTAAAGTAAATAAGAATTAAGATATCTATAAAGGCATTCTTT

AACGTAATAAAGGCTTCTCTACCTCTATCTCCTATATCTATAAGTACGCTTTCCTCTTTTTACTAAGCCT

AACCTCCTTTAGCTAAACTAGCTTTCTTCTTTATTAATTCTATTAGTAGAAGTATAATTTTAGAGAATTT

AAGTATAAAATATCTATAGTAGTTTATAAAACTTAGAAATACTCTAATATTATAAATAGTATATAGTTTT

AGCTATTCTTAGGCTATTTTAGCCTAATTTAAGTTAATACTAATCTCTTTAGAAAAAATAATATAACTTA

GGAACTTAGTACGCTATATATACTATTTATATTTTAAAATCTATAAATATAACTTAGGTTTAAGTAATCT

TATTAGGACTTCTTTTATATAGTATATATATTCTTTTTTTATATTTAAGAAGATTAAAATATTATCTAAA

TATATAATATAGGTTATATCTACTAATCTTATAAAAATATAGTAAATATATACTTAAATTACGCTAAAGT

ATTTATTAAGCTAAAAAGTATTATAGTATACTTAAAATAGCTATATCTTATATAGAAAGTAGTCTTTTAT

TCTTCTCCCTTCTTAATTTATACTTAATAATATACTTCTTATATATTAAGCTTTATATAGTACTTAGCTT

AGGCTAACTTATCTAGAGACTTAGTAATTAATAGTAAAAGATAGTAGTTTTTAATAGTTATTTTATTAAG

ACCTCTATAGTCTATATATAATCTTATACTTTTATCTTTCTTCTTAGTAAAGAGAATTAGTATACCTACT

AGTAACTTTAAGTATTTAATCTACTCTCTTATAAGATATTCTTCTAAATACTTTTATAATAGCCTAAGTT

CTATCTTTAAAAAACTATATAATAGACTATAGAGTAATATAGTACCTTTTTATAAGGTTATTACTAAATT

ATATAGACTATATTTAGTAAAAGAGTATAACTTTACTTCTAAGTATAGCTCTATAAACTTAGTATACTAT

AAGAGTAACTAAGGTAGTATACCTCTATCTAGGCTAAACTAGGCTTTATTAGCCCTTTCTAGGTTAGCTT

TATCTTTATTATCTTTATTAGGGTTAAATCTAGCTATAGTAGCTACTAAGAGCGCGTAGATATTTATAGA

GAGTATTCTAAGCGTCTTATTAAATTTCTTAGCATTTTCTATACTAATTTTCTAGAATTTTAGCCTTAAT

ATATCTTTCTTACTTTTAAAAAGTACTCGCCTTATTATATAGGTAATCTTTAGTTAGTATATATTAATCT

AGAGAAGACCTAAATAAATCTTAAAATATTTAATCTCTCTAATAATAAAAGAGACTACTTATAATCTAGT

ATATTTTTATAAGTTTATTATCTTTATAGTTACTATTATTATACTATATAACTAGATCTTTAAGTCTCCT

AAACCTTCTACTAAAAGATTTAATATAGAAGTAATACGTAAGTTCTATTTCTTTACTAAAAGAGCTAATA

GAAAGTTTAACTATAATCCTCTATTTATAAAGACTTCTATTAGATTTAATAATCTAATTTAGGCTATTAC

TATTAGTAGTAGAGAAGGTTCTCCTTATAGTAATTCCCTTAAAGACTTAGTATCCTTTATTTTAGCTAGT

TAGTAGATATAAGTAGAAAGTATAGTTATTATACTTTCTACCTCTTACTTTTTCTTACCTTATTCTCTAT

TATATTAGAGGGGTTCTTATTATTTTATTTATAGTCCTTCTACTTATATTTAGGTTTCCTATACTTAAAA

TAGATAAAAGTAGAATATTTACTCTTATCTTAGGCTAACTTAGGCTAATTTAAGCCTTTTTATTCTATTA

TATTTTACTTAGGCTTTTTCTAGATCTTTTAAGTTTCCCTCTCTTCTTCTATAGAGAATTTCTTCTAGTT

CTACTTCTTTCTAGACTATTTTATAGATATTAGTTTATCTCTATAGTTTCTATCTTAAATAAGCTTAGTA

TAGCTATTAAGGTTACTTACTATACTATATAAGCTTAGATTAAGAAGTAATAACTCTTTCTAGACTATAG

AGACTAATACCTCTATAAAGTCTATAATTCTTTATTTCTCTAAGTAGAAAGGTCTTAGTTCTATCTATAG

AGTGTCTAACTTTAATAAAAGGTTATTTAGATTCTAAGTTTACTTCTACTTTATACTTTTTAGGTCCCTA

TAAGCTATAAGCTTATATTATTCTATAGAGCTAAGGGCCTCTAATATATTTACTTTTAATTATAGCTAGG

AGAGCTACTAATATAGGTCCTTTCTTTAGTAATTATAAATATATATAACCTAAGTTATCTTAAGAGGGTT

TAAGATAAATTATAGTTTAAAATTAACTTTAACTATTTCTATTATAAAGATTTAGGGGTTTTCTCTAAAA

GTTACTTTATATTCCTTTTTCTATTAGTTAAATTTAGCTTAATCGCGGTTTATAAACTATATTAGCTTAT

CTAGTGCTAGTGCTCCTATATTTAAGGTAATATAATATTTAATAGGTTAAAAAGGGGCTTAGAGGTCTTC

TTATAGTATAGTAAAGTCTCTTTATTCTATTCTTTACTTTAACTCCTAAAGATAGGCTAGTTCTTACTCT

TTTAGCATTCTCTAGCGCTATTTACGTAGTTCTAGAAGTTAAAGGTATATTTCTACTAAAGAGGTCTTAG

ACTAAGCTTCTTTAGTATAACTTAAGTTAGATTAGGTCCCTCTAGAGTTATCTAGTCCTTCTATAGTAGC

TTAAGTAGGTTCTATTATAAAAGGTTATAACTATACCTCTAGGGATCTAGTTATCGCTAGTCTACTATAG

TAGTATATACTTCTATTATTAGTAGTACTTATTACTCCTCTTAGTTATACTTTAGGAAGAGCTATTTAAA

TATAAGACTATTAGGTTAGAATAGGCATAGGTAGAGAGTAGTACGAGTAGTTAGAGAGTAAATAGTCTAT

ATTTAATTAGATAAACAGCTACTAAGCTAAGGTAGTATTTAGAGAGCTTATATAAGTAGCCCTAGATTAG

AGCTGACTAAGAGGGGCTAGAGTAGACTAGCCTAAACCGAGGTGGTGAAGAGGTCTACGAAATAGAACGG

TTCCTAACAGTTAAAGTATTATATAGAGTTTAGTGCTAATACTTCTCTAAGCTTAATCTAGGTTAGGGCG

AGTGAGATAGTGTAAAGAGCGACATAAGGTAGTTATTTAGCAATATAGTAGAGTAGGCGTGCTTAAATAG

AAAGTAATAATAGGTTAATAATTCCTAATCTATATTAAATAGACTAATAAGTATTAATCTTGTGAAGTTA

AGTAACTAGTAAGAAAGTACAATTTAAGTTGACTACAACGAGAGTAATAATAAAATCTATCTATCCTTAT

ATCCTTATTACTTCTAGTCCTGCTATCTCGTCTATCTCCCCTTAGTAAGATGGTAGAGATGGTACTGCTA

TCTCTCGGCGCCCTGCTATCTTAGATGGCTGGCCTTGGCGAGATGGCGTATTACGTGACCTCGAGCCCCG

TGATGATCCATTACCTTTTGACGTGGACGTCCCTTGGCCTTTAGCCGTCTCGTTATTAGTTAAGGAGAGA

ATCCTTCTTGTTACTTGTAACACAACTCTAAGCTTCTTAGAATTTGTCTTTAATAGCCCAGCCTATAACA

CATGGTACTTCTTGGCTTACATGTTAATTAGAGGAAACCAAAATTATAAACATTAATAATCAAGCTAAAG

CAACACCAGTCCATGCTCTATCTTCCTGGAAAACCAATCTTACTTTATCGCTGCCGTCAAGCTTGCACCT

ACAGATTGAACCACCCAGATCTGCTACATAGACGTGTTCGTTTCGCGCATCAATCTTGAGCCCAATTGCC

TCGTGGAACTTCCTAGCGATGATCTCGTGTTTGAGGTGCGGTGTGCTATCAGTGTGTAGAGCATATCCAC

GATCATCAAACCGCAATCGATTGAGAGTGTTGCCGAAGGGCATTTCTCCCCTGTCGGTCCAGTACAGGGT

TTTGGTCGATTCGTGAAAATCCAGATCAATTGGTTCAGCAAGCCCTTCAAGAAGACAGACCTTGTCCTTC

CTATGAGCGGCTGTTTCGCCGGGCGGTATGGTCATATTTGCACTGAAAATACGACCCTGCCAGCCTTTTG

ATGCTCCTTTTTGCGTCCAAAAGATTTTCCCTAGGGTGTGTGAAAAAGTGATGCCAACACACCATCTAGT

AGCATCGCGTCGATCGCATTCGTTGTTGTTGTCCCCCGTCACGACGACCTGTTCAAGGTTGCTTCCATCT

TTGTCGCAGCGCCAGATACAAAGACCCTCGCGATCTGTGAAGTAGAGTTTCTTGTTTGCTTCGTCGATGA

CTATTTGCTTTGGCGTATGAACGATTCCTTGTTGGATAAGTGGTCGAATATCATTACCATCCAGTTTTCC

AGCATATATCATGCCGTCTTTTTGTCCTGGGGAACCCATACATGTCCAGAACAGGACGTTTTCTTCTTGA

GACACCGCAATACCATCTGGTAGGTACTGGTCCTTAAATACTGTTCGAATATATTCGCCTTCAAATGAAT

ACTCGAGTATCTTGCCTTGCTTCAAATTGTCGATTTGACCTGATAAGCCATTGTCTAATACAAATATTCT

AGGCTTCGTGGACACGGCATCTGCTGCTGTAGAGGGTGGATAGAAACCACCTTTGTTGCTCTTAATCCCT

AGTTTACCCACGTCGATGAATTCCCTTTGCAGGTAGTCAACAGTGTATGTTGTGTCTAAATGACGTTCTT

GTGCGTATGTGCGCTCGATGTTCGCTACCGTGTCGAGGCCAACATCTGCTTATAAGTTATTTATCAATGG

GATCGTTATTACAAAATAATTAACTCACAATCCATAGCTGCGAATGGTCGAGTGCCCGGTCGCACTATTG

TCTCAACGAAAATGTCATCAGCGGTTTGTGCGTCGACAATTCCTTCTGCCACTACATGTAGCATTTCACG

TTTGATTGAAGCCCATACTCTGTTCTGAATAAATCCGGTCGATTCCCTCTTTGCCACAAAAGGCTTTAGC

CCAACTGTTTTCATGCGATCAACAAGCAAAGACATGATTTCTGGGGCTGTATTTCCACTCGTCATGAGCT

CAACAACACGCACATGAGGAGGCATGAAGTAGTGCGTATTGAGTGTGCGTCTCTTTGCCGAAGGAGTCAT

CTTCTGCGACACCCAAGATGACCTATACGACGAAGAGTTTGTTGATAGTATACTGTCTTGTGCACACAAT

TCTTCAAGTTGCGTAAAAATATCCTGTTTGAGTTGTAAGTTCTCCGGAACACACTCGATTATGTGCCAGG

CGTTGGAAACGGCACTTTGAAGATCTGCAAACGGGATTATCTGAGCCGGGGGTTTTGCCGTTTCTAAGTG

ATATATTGACTTCTTTTCATGGAAGTATTTCGTAGCCTCAATGCGTTGGTTCGAATCTGTGTCATATAGG

TGGACTGTGTAACCATTTGCTGCCCAGCATGCGGCTAAGTCTGTCAGATGTGCTACTACGCTAAAGAGAA

TATATACGAAGGAAATTGAAAGTTACGCAGACGACATAACCTACCAATGCGCCGTCCAAGTATTCCAGCT

CCAACCACTGTTACAGGACGGTTTTCATCCCCAAAAGCCTCTTGTGATGTAATGAGCCGACTCATAACGT

TTACTAGACTTGAATATAAAAAACCAATGAGAATCCTTATGTTTATAGGGTTTTTTGCATATTTTATATT

TCCAAGAGTTTTTGATGTATATGGGTGATGACTCAGGGTATAACAAGGTGAGGTATTTTAGCTAATGTGT

ATTTAGCGTACGGAGCAAATAGTATCAAAAATAACCGCCACTACCTGACCTTACTCAAAAAAAATAATGG

GGTGCAGGTTTAGCTCCAGACATAATTATAGCTATAGTAATCACTTCGGTCCCCGTCAAGATGACGCTAT

AGATTAAGTCATAGCTAGAATACATGAGACAGGACCATAGAAAGAAGAATTTTATAAAAGGTCTATCCCT

ACGTGCTAAAAAAAACTCTATATACTACAAACGACCAATCGGATCGCGACGTTTAGAGGGAACTTCGTGA

TTTTTGTGATCCGATTGGTCGTTTTTAGTATACAGAGATTTTTAGCACGTAGGGATAGACCTTACTATAC

ATATCTACTAAAAGAAGACTGTATGAGATTGTATTATGAATCGCTATAATATTCGTCGCGATGTAAGGCG

TGAAGATTTTGGTATTCTGATCACCCCATACCAGATCGTGAGACTTTTGCCACTCTATTAATACCCATAT

GTAATACTAGAATATAGATACTATTCAATACTGTCTACCAATACTGGAAAAGGGCGTTTGTTGTGTAATA

GAGGGTTATTACAGAGAGGACCTTGGGAATTAGATAGGCTACTAAGCAAGCTGTTTTCAGGTTGTGAGGG

GTAGTCAAGGGCCATTTATGATGATAATAATTGTATTGATGATTTTCCTTCCTAGAGACGCGACCGCGTG

CCTCCTTAAAGGAGTCTGTTTGCTTATTCTGACCTCAGCCACCTCTTCTTCGAGCTGGGGTAGGGATTAG

CCTTGGCGGCTCGTCTGATTGTCTGTATACAGACTCCGGGTGCTCAGGTCTACACCCGGTATCTGCTGCC

CAGTATCTGACAGCTGTTTGAATTTTGACCGGTAGACGCCGAAACAGTGCGGATGTTGCTAGTGGGATAC

GTAGTAGGAAACACCATAGATGTCACAGTAAGACCTATACAATACCTCCAATACACATTAATAACTTGGC

GCGTCATGACTTAAGTAGTTCCTAAGCATTATTAACCTGTTTTTTGTGGCGGCCTCGGCGAGGTCAACCG

TGCATACCTACCCTGCTTTTATCGCGCTACGCGTCTTACTACTAACGCCAAAATCAGCTCTTTAGAGCTG

ATGTAGATAAAGGAGAAAATAGAAAAAAAATGATTAATCACTATATGTGAATGTTACTATGTCACATTCT

ATTGAATCATAAAATTGTCGCCTATCCGCACTTTGAGTGTCATTAGACTTGTTTTTCTAGCCGCGGCCTC

CTATAGGTTAAGGATATTGTGATAACATTCCTAGTAATATCCTTCCTTTCTGATTGAATTTCTAACAATA

CTGAATATTTCTTACGGTTAATTATCAAGATATTACGTTTTATTTCATCCCAGGGTTAGCTCGCTGTGTA

GTTTCCAATGGGAGTGACGTAGGGTAGCGCGGATTAACGTCTGTGCGATGTCAGCTGTTGGCTACGCCAG

TCATGCCGTGCATATAGCTGCGGAGAAAACGCGGCTGAATAAGGGTAGGACAAATGTAATCTGATCTATG

GTTTCAGATGGTGTGAATAGTAAAATAGCATTATTACTTGATTAGATAAGCTCTAAAAAGCCTAAAGCAC

CATACTATCAGTATTTTATATAAACAGAGCAACTCTACCCAATCTCCGCTCAAATATCCACCTCTTCTCG

TTACGCCGACCATCGTACATCGTGCCGCACCATGACTTCACGTCAGAACACTAACACGCCAATGCCTTTG

GCCATTATTGGCATGTCTTGCCGCTTTCCTGGGAAAGTTGCCTCTTTAGAAGACTTTTGGGATATGTTGA

GCAATTCTAAGCATGGATATCGCCAATTTCCGAGAGAACGTTTCAACTGGGAAGCATTTTACCATCCAAA

CCAGTCCCGCAAGGATTGCATCGATGTCAACTGTGGTTATTTTCTTGATGGTGATATCGCCGAATTCGAT

GCACAGTTTTTTAAAATGAATGGTACAGATGCAGCTTCTTTCGTACGTCTATAAATGTAGTTACAGAGTG

ACAACGCACCTGTCCACTAACCTACTGTAGGACCCACAAGGACGTATGATTCTCGAGTGTGTGTACGAAG

CCCTAGAAAACGCAGGTGTTCCCAAAGAGAGCATCGTTGGCTCTAAAGTCGGGGTGTTTTCTACATCTAA

CACTTCTGACTACACTCTGTCGCTCAAGGATGACATATATTCAATGCCGGCACTAGTAGGCGTCCTCGGC

CACGCCTGCATGCTTTCGAACATTGTCTCCAACACGTTTGACCTAAAAGGTCCTAGTGTCAGCATTGACA

CAGCATGCTCTTCAGCCTTTTACGCGCTTCAGTTAGCATCACAAAGTCTCAGATCTGGTGAGACGGAAAT

GTGTATCGTTTCTGGATGCGCATTAAACATATCGCCTTGGCGGTGGACCATGTTGTCAAACCTGACGATG

CTCAATCCGGACGGTCTAAGCAAATCCTTTGATCCCCAGGCTGACGCTGGTTATGTCCGTGGGGAAGGCG

CAGCAAGTATCATTGTTAAACCACTCGATGCTGCGATACGCGACAATGATCGGGTGCATTGCGTACTATC

GGATATTGGTGTAAATCATAACGGCCGCACGAATGGCTACACCCTACCTGATGCAAGGATGCAAGCTAGT

TTAATGAGAGAGTTACAAGTCAGACTGGATATTAAGCCAGATGAGTTTGGTTTTGTGGAAGCCCATGCGC

CCGGCACCCGAGTTGGAGACCCTATTGAGATATCAGCACTCCAAGAAGTATTTTCGACTTCAGCACGTAC

CCTGGAAGACCCACTTTTGATTGGGTCTGTTAAGGCCAACGTGGGACATCTGGAGTCCTCTAGCGGCTTT

CCCTCGCTTATCAAAGCTGCCATGATGCTGAAGAAAGGTCTTGTTGTGCCAAACGCTAACTTTGAGAATG

AATCGATGAACTCACACTTGAAGGAGAAAAACATGAGGGTAAGTCTAAAATACTTAATAAAATTGTAGAG

GTATGACGTTTTAACCTTGGCTAGGTACCAATATCTACACAGCCTTGGCCGAAGGGCAAGACATATATAG

CCATTAACAATTACGGGTTCGGCGGATCTAATTCTCACTGTATCGTGAGGGCGCCGCCTATTCCACAGGG

GTTGGTGAGCCAAAAAGAGACTCGAAATGTTGAGAGCGACTATCTTTTTGTTCTATCTGCCAACGACGAG

GTCGCCCTCAGACGAACTAGGGAGCAACTGGTTGAATTCCTTGAATCGGTTGACGCGTCTTCCACAACCA

TGCAAAACACCGCATATACACTAGGGCAGCGTCGCTCTCTCCTTAGTTGGCGTGCTACCGTTGTAGCCTC

AAACATTGACGATCTCATCATTCAGGCTGCCTCCCCACAGGTTATTCCCCGGCGGGTCACCCGTCAGCCA

ACGCTAGTCTTTGCATTCACGGGACAAGGTGCCCAATACTTTGGTGTGGGTCGTGAATTACTCCAGTATC

CCGTATTCTCTACGACTCTGAAGATGGCTTCGGCCTGCGCTGAGTCTTTTGGTGCCAATTTCTCGCTCCA

GGACGAGCTTTATGGGAACGAGGCAACGTCACGGATTAATGACGCTGATGTCAGCCAGCCAGCTTCAACA

GCTATCCAGATTGCTCTTGTGGACCTTTTGCGCTCATGGGGCATTCAGCCTTCAGCAGTTGTAGGTCATT

CGAGTGGTGAAGTCGCCGCGGCATACGCCGCAGGCCTCCTCTCACTCCCAGGAGCAATGCGCATTGCTTA

TGCTCGTGGTCAAATGGCTATCAGGATTAAAAAAGTTCAGCCAGACTTCAAAGGTGGCATGTTAGCGGTT

GCTGCAGGGCCTGCTGACGTGTTACCGTTACTGGACATTGTTACCTCTGGTAAAGTTGTAATTGCCTGCG

AAAATTCACCAAAGTCAGTTACCGTCTCTGGAGACGAAGCCGGATTGGTCGAACTAGAATCACTGCTTGA

AGAAGATGGACTACCTCACAGGCGGCTAGCTGTGGATTTCCCCTATCATTCAACTTTCCTGGATCCATTC

ATCGACGATTACGAGGAAGCTATATGCACAGATGATACGTTTTCCAACTTGCAACCCACAGCAGAATACT

TCTCTGCCATGGCAGGAAGGAAGGTTGAACCTGTCACAGTACAGAAACCATCGTACTGGGCGAGCAGTGC

AAAATTTCGAGTGCGGTTCACATCCGCCGCAAAAGCCCTGTTGAGGAGCAAACCTTCGCCCAACGTCGTT

GTGGAGATAGGCCCGAACCCTACGTTAGTGGGGTCCCTCAAGAGCATTTTGAGTGAAATCAAAAAAGAAA

TACCACATCCAATTGAAGTGGTACCATCTCTACACCGCGGCCAGAATGCACGAACCGCAATGCTTAAGCT

TGGGGCTTCTCTTGTTAGTTTTGGTCAGCGTATAGATATGGAACAAGTCAATTTTGCATCAGGACATATC

AGCGGCCAGCCACCGACGCTTGTTGATGGCATCAAGCCGTACCCGTGGACACGAAGCCACCACTGGATCA

AGTCTAGGGTCCGTGACGATGATTTGCATAGACCATTTCCACACCACGACCTCCTAGGCTCGATCAATTC

ATCGTGGGGCAGTAAGGAGTTGGTCTGGAAAAACAACCTCGACGTAGAAAACGTGCCGTGGCTTAGAGAC

TATCAAGTTGCATCATCCATTACCTACCCTCTAGCTGGTTATGTATGCGCTGCTATTGAAGCCAGCAAGC

AGTTTGCGATGACAAGAAATCTTTTCCTCGACAGAGCATTCAAAGGATTTACCGTTCGTGACATGATTAT

AGACGAAAGCCTCGTTATGAAGGAGGGTATACCCGTTGAGTTGGTCACCAAACTTCGCTCGCTGCCTGGT

ACCAACTTTGAGGAGTTCGAAGTCTTATCCTGGGATGAGGGCCAGCGAGCCTGGAAGAGATGCTGCCGAG

CTCTTGTGAAGTGCGAAGCGACAACAGATGGAGTTGAACAAGTGGAAGAAATGAAATGGGCTGAGTCGCG

TGCGGCTTGCCACAGCTGTGTGGGCAGCCCATTGCTCTACCAGAGATTATCAAAGGTCGGCCCACGGCGC

ACTGGTAAATTCCGCAATGTGGTTGACCTACGTTATGGAGCTGGTAAGACGACTGCCGAAGTCGTTGTCT

CCGACACCAAGGCAAGCATGCCTCAGCATTATGAAAGCGACATGACAGTTCATCCGACAACGATTGATGG

CCTGTTCCAGTGTGGAAGTTGTATACCTTTTCTGGATGAATCAAGTTCTGTTGTTGGTGGCTCCAGCAAT

ATATGGGTTCCAAGATCTATCAAAGAGTTCACGATCCAAACTCGCCCTGGAGAGGCGCTAAAACCAGAAA

TGGTTTTTCGCACGGTCGCTCGGGTAGACAAGAATGAACGCCACGACAGGTCATATAGTATCGACGGGAC

CACAGATAATGCCCCCATATGTCAGATACGCATTCGAGGCCTCAAGTTGGCTGTTGAAGCGACACTAGCA

CCTCAATGGCCGGCGCCGCATTACGGGTGCTACAAGATAGCCTGGCAAAACGCTACGGAGCTTCGGTCCC

AGGCCGCCCAGTGGCATGTCCTACAGGGACCAGGAGACGTCAAGAATTTGGCCGGTAGTGTCAGCAAGAA

AATCGGGGGCACTGTTCGGCCTTTGTGCGAAGGTGTGCCTAGTGAGGCCAGCTTCTGTGTCGTGGTGGAT

GTGGGAGAGGGGCTCCTCGCTAGTGTGGAGCGGGAGTCGTTCAATCATATCAAACAGGCGCTCACAACCT

GCGAGGGGGTACTTTGGGTGACATGTGGCGCCTTTGGGGTGTCTTACGATTCTACACACCCTAATGCGGG

CATGGTGACTGGCCTACTTTGGACCATTCGCAGCGAAATGCGAGCATCAGTAGCATCACTTGACTTGGAT

GCCAACGCTAGCAGTGACATTGAAGCACAGGCGGCACTTGTTAAGCGTGTAGCCGATCACTTGGCAGCGG

CTGCTCAAAACGCGGATGTACAGGCTGAGATGGAATTCACTGAAAAGCAGGGCCAGCTTATGGTATCGCG

CGTTGTGCATGATACACAGCTGGATAATGTTGTTCACGCTGTCACAGGAGTCATCGCGCCGCGCACCGAG

CCCTTCGACCCGGAAGTCAGGGGCTTCTTTACCCTGCAGCGTCCAGGTATGCCCGATTCGTTGTATTTAC

AACGTACCGACGTTCCGGACCCGCTTGATGAGAGTGAGGTTGAAGTACGCATTGCAGCGATAGCTCTCGA

CGCCGATGACATCCACGGATTGCAGGGGCGTGCTCTGAGCGGAACCGTTGTGAGATGTGGATCCACAGTT

ACACGTGTGCAGCCTGGTGATCGCGTCTTTGGTCTAGCAAATATCGATGGCGCAGTCCGAACCTTTGCAC

GTGCGCCTGAGACATGTCTAGCCCGCACGCCTGCGAATATCCCCATCGATGCTGCTGCGGCGCTTCCTGC

TACCCTTGGAGCTGCCTACCACGCCCTCGTTGACCTCGGCCGACTCGTCGCGGGCGAATCTGTCCTCATC

GTCGCAGTGGGATCTGCCCTTGGCCAGGCTGCAATTCAAGTTGCGTTGGCAAAAGGTGCCCTTGTCTTCG

CCCTTGCTCATTCACAAGAAGAGCGCGACGCTGCCATTGTTGCGGGCGCTTCCATCGACCGGGTTGTAAC

CACTTTAGTGGGGCTTCCGCCTATCCAGATTCTGTTCAACCCCGTCTCAGATGCCAATGCCAACCTTAGC

ATGTTGGGGGCCTTGGCACCTCTAGGACGCATTGTGCAAGTGGGGGAGCCATCACACCAGTACCCTGCCC

TTGCGGTAGGCCACAGTTTCTCTATAGCACACTTGGATGCTGTAGCTGATGCTCTGCCCGCGCAAATGGC

TGCCATACTTGATGCTGTGGTAGGGTTAGTTGACAGCAAGTTTGTGCACAGTCCTCCAGTGCGGACTGTT

GGGCTCGAATATTTGTCAGAAGCCCTATCCAATATTTCGGAGACCGACTCGAAAAAGCTTCTTTTGGTTC

CTGGAAAGAATGAGATGGTAAAGGCTACCCCGTCCTGTCCTGCTCCACCCACGTTTGATCCAGCGGCTGT

GTACCTGCTTGTCGGTGGTAGCGGTGGTTTAGGTCGTGTCATCGCAAAATGGATGTTAAACAATGGAGCT

AGAAAAATTGGGCTACTCTCACGCAGCACCTCTATGAGCCCTGATGTTCGCACATTAGTGGATGATGCTG

CTGGGATAGGAGCTGAAGTTTTCTTGTTACCGTGTGATGTCACGAGCCAGCACCATTTGCAGCGCGTCAT

TGACCAGTGCGTCATCGAGAAAGGCCAAATTAAGGGTGTAATCAACGCGGCAATGGTTTTCAAGGTAACC

CTTCTCCTAGTCTGTTCGAACTCTTCAAACCCTCCGCCCTCGGCCTAATCTCTTCAATGTTTGGTGCTAA

TTTTATACGCAGGGCGGAGTCTTTACCTCGGTTTCATTCGACGATTTTACTTCTGTGGTCCAGCCAAAGG

TCTGTGGAACATGGAATTTGCATCATGCTTTGCGTGAGGCAACTCTAGACTTTTTCATTCTAATATCTTC

GGTGGCTGGCATTATGGGCACACCTGGGCATTCAGCTTATGCCTCTGCCAATACCTTCCTGGACTCTTTT

GCAATGTACCGCATGCAGCAGGGACTACCGGCTACCTCTCTAGCCTTGACTGCGGTGGTAGACGCTGGCT

ACATGGCTGAGAATGCTTCCAAACTCCAAAAACTTAAGTATGTTAGCGAATTTGAGGGCGAGATTCTCCT

CACTGCTGATGTACTGGCTCTCCTTGGCGCTGCGGTCACAGGATCAATAGCTTCAAGCTGCAAGGGTTTC

TCTATAATCGGGGCAGGCTTTGGGACTGCGCTCAAGCTGCCCTCATATGCCCAAGATCCCAGATTCTCGA

CCCTGACATCCAATCACTCCCAAGACCGAAAATCGAAGCCGAGAACAACAACTGCGGCCAATACGGATAC

ATTGGTTTATGCAGTGGACCAAGCTGACACCAAAGAAGAAGCAACTCAGTTACTATTAGCTGCCATTAGA

GATAAGATAGCTCAGTTGCAATTAATTCCTGTTTCAGATATCGTCGACGATCAGACCATCACGGAACTCG

GACTTGACAGCTTAACAGTAATGGAGTTGTATTCTTGGGTAGGAAGGTTGTTCCGATTGAGAT

TTGGAATACAAGAGTACGCAAGGTTAGACACCCTGGAGAAAATCGTGGACAGTGTGATAGTAA

AGCGAGAGGCTGCTAAAGTCGAGGCTCCATGATATGACCATAGATCCTCATAATGTTATAGTT

CAAGCAACCGTGTTTTGCAGTCCATATTTGTAAGCCTAGAATTACCTTGTATCGTCAAGTGCT

TGTGAACTAACTAATAATAATTCAATTAAACTTACTCTTAGCATCAGATACAACACAATAGAC

AATACTTCTTACATGCACAGCTACTGACATTGAACATACTGTAGTGGGACATCACTGGTAGCT

CGCGGAGTGAACTTAGCATCTACTGGAGTCTTGTGCATAGGTGCAAAAGTTGATTCCTTTATG

AAGAGCCCATTAACATCCCCCCTGTGTGACGCAAATTCCTTCAAACCCGAAGAATTGGCGATT

TTAGTATCTTAGAACGCCTAGTCTCGCTAGTATTATACTAAAATCAACAAACTCCTTTTCTTA

ACGCTATTTTCACTAGATGCGAGAGGGGTATTGTGATAGCACTATATAGGGAGATAATCAGAT

TATTACCTGTAAGTAAGATACTGCGTGAGGAGTACGCTGGTAAATAGGTACCCCCTACCCGCG

GGTTATGTAGGGGTTGGGTTAGGTCGCACGGGCCACGGGTCGGTGTCGGTAGTAGGTTGCTAG

GGCTTAGGTCGGTATCGGTGGTAGGTTGCTAGGGCTTAGGTCGGTGTCGTGTTACACGGGGTT

TTAGGTCTATACTGCTCCGGTTAGGTCCGCCACTATAGCCGATAAGCCTTACGTTAGTGTTTC

GTGAAGTTTGTCTAGCGAGACAGTAGAATAGTATACTTAAGATAGAAAGGTAATAAAAGGTTA

GATAACTTAATCTATATTACTATAGACTAATAGGTAATAATCTTATTAAGTAAAGAAACTAGT

AAGAAAGTATAATTTAAGTTAACTATAACGAGAGTAATAATAAAATCTATCTATCCTTATATC

CTTATTACTTTAGTCCTAGTATCTAGCTAAACCGCTAGTATGCTTAGATGTTGTTAGTAGCTC

TAGATATCCTAGTATCTCTAGAGAACCTAGTATCTCTAGATATTCTATCTGTTACAAGTAACA

AGAAGGATTCTCTCCTTGACGAAGGGATAGGAGGCAAGGAGCCTATGGAACGTCCACGTCAGA

AGGTAATAATTAACGCGGGGCGTAGATCACGTAACTTAGCATCTTGCTAAGATAGGACATCTA

GAGATGCTAGGTCCTCTAGAGATACCAAAACATCTAGAGATGCTAACGACATCTAAGATACTA

GCAGCTTAGCTAGATACTAGGACTAAAGTAATAAGGATATAAGGATAGATAGATTTTATTATT

TACTCTTATTATAGTTAACTTAAATTGTACTTTCTTACTAGTATCTTTACTTAATAAGATTAT

TACCTATTAGTCTATAGTAATATAGATTAAGTTATCTAACCTTTTATTACCTTTCTATCTAAG

TATACTACTCTACTATCTCGCTAGATAAACTTTACGAAACATTAACGTAAGGCTTATTAGCTA

TAGTAGCGGACCTAACTAGAGTAGTATAGACCTAAACCCTATATAACACTTTAACTACCTATA

GATTTAGTAGACTTAGAAAGATTAATATTAGGACGCTTAATTAAGATTTAAAGATGTCTTACA

ACTAGGCTTAGTTATAGGTAGGTACTAGTAGACTAGTCTAGAGTCCTAGACTAGTGCCTACTT

TAGTAATTAACGATAAGATGCTAACCCTAAGTAATAATAAGGTAGATAATAACGTATATAACT

TACGAGAGTTATAGAATCTTAGGCACTAGGCTATAATCTATATATAGTAGATAGCTAAGATAT

AAGTAATAATTAATTAGCTTATAAGTTAGCTAATAGCTATACTAAATAAAAAGCCTATTAAGA

AACCTAAGATAGCAACCCTAGATAAATACGATAGTAACTAAGAAGGGTTACAGACGTTCCTTA

CTAATATTAAGTTATACTATAGCTATAACAATATACTAAATAATAAAGAAAAGATACTTATAG

CTAACACTTATATAAAGGGAAAAGTAGTAAGCTAGATATAACCTTATATAGAGGACTTTTTAA

TAAATATTAATAATAAAGGAACAAAGGATAAGATATATACCTTATTTAGTAGTTAGGCTAACT

TTAAAGAGGAATTAGAACGTATCTTTAGGGAGGTAGACGTAGAAAGCTAAGTGGAGAAGGCTA

TATCTCGCCTAAAGTAAACTAAAGGAGTTTTAGTTTATATAGTAGAGTTTAAGTAGCTATAAG

TATAAATTAATTAAGATAATTTAGCACTCTAGATAGTATACGAAGTAGGTCTAAAGGAAAATA

TTAAGAACGAACTTATACACTATAATAAGCTAAAGAATTTATACTCGTTAATTAAGTTAGTAA

TAAGAATTAATACGTAATTATAGGAACGAAAGAAAGCCTAGAAAAGATATAGACTAAGGCCAC

TCTTTACTAATATCTAAAAGTATTAGAGTAATAAGGATTATAATAAAGATATATATATAACTA

GTAAAGTATAAGATAAGTCTAAGGATAAGAATAAAGCCTAAAGAAAGTTTAATAATAGTCTTT

CTAAGGAAGAATACTAGAAGCGTTATAATAGTAAGGTATGCCTCCACTACAGCGAAGTAGAAC

ACTTCTATAGAGACTACTTAAAGAATAAAGTTAAGTAAGAAGTAGTTAAGATTAAGATACTAT

AAATAGTAATACTATACCTAACTAAGGAGCTAGATAAGACTCTTAGCGACCTAAACCTATATA

ATAAAGTAAGATAAGCTACTAATAAGGCCTTTAAGCTAGTATAGAAGGTAATAAGGCTCTAAA

ACTTTAAGTAAGATAAACTACTACTAATAGATTAGTAGGTTAAAGGGGTAGAAGTACTACGCC

GCCTAAGACACTAATAGTACTAGATTTATAGAAATACTAGGCACTAAGCTAATAATTATAATA

TTAAAGAACAAATTATAATTATAGGACCTTATATAGAAGAGATTATCTATAGAGCTACTTAGA

AATAACCTAGGTTTAAGGAATTTAATAATAAGAAGACGCCTTAGATATTTAGAGAACGAATAG

AGTAATATAAGTACCTACGTTAGGTTAACTACTTAAGATAGTACAAATATTACTTAAGGAAGT

AAGAAGAAACTAGAAATAATAAGGATAATTGCTACTATATTAACTTATAGTATAATAAGTATT

AAGTATAGTATTATTAAATATACTAGCTAGAAAAAAAGGAAGCTTATAGGGAGTTATACTAGA

TCTATTATATAACTAATTACTAGTCTTATAAGGAATAACAACGGAATACTTAAAAGGTAAATA

ACTACTATTATAGTATAATATCCACTAAGAAATATAATATAAAGAACTATAAGATATATAGAG

CTATAAGACTAAGAGTTATAATAACTAGAGTACCTAAGTAGAGTTAGAAAAAGTCTAAGAAAG

AAAAGAGCTACAAACGACTCTTATAGACGCATTATAAGAAAGAATACGCCTTCTACTAATAAT

AGTTTAAAGATACTTAAGTGTTACAAGTAACAAGAAGGATTCTCTCCTTGACAAATGACGAGA

TGGCTGAGAGCCTATGGACGTCCACGTCAGAAGGGAATGATCAACGCGGGGCGCTGATCACGT

GACTTGGCATCTCGCCAAGACAGGACA

Figure 1. Transposon fragment containing an intact LTR found upstream from the LAM1 gene in the genome of *C. heterostrophus* race T (Scaffold 4LU). Highlighted in red and green are sequences related to LAM1 and PKS2, respectively. Highlighted in blue are sequences with high identity with LTR (Long Terminal Repeat) of transposons of the *Gypsy-3_CH* family. Sequences in yellow have identity with sequences of transposable elements.
